# A Critical Evaluation of Validation and Clinical Experience Studies in Non-Invasive Prenatal Testing for Trisomies 21, 18, and 13 and Monosomy X

**DOI:** 10.3390/jcm11164760

**Published:** 2022-08-15

**Authors:** Zachary Demko, Brittany Prigmore, Peter Benn

**Affiliations:** 1 Natera, Inc., San Carlos, CA 94070, USA; 2 Department of Genetics and Genome Sciences, UConn Health, Farmington, CT 06030, USA

**Keywords:** NIPT, trisomy, massively parallel shotgun sequencing, digital analysis of selected regions, single nucleotide polymorphism, cell-free DNA, meta-analysis

## Abstract

Non-invasive prenatal testing (NIPT) for trisomies 21, 18, 13 and monosomy X is widely utilized with massively parallel shotgun sequencing (MPSS), digital analysis of selected regions (DANSR), and single nucleotide polymorphism (SNP) analyses being the most widely reported methods. We searched the literature to find all NIPT clinical validation and clinical experience studies between January 2011 and January 2022. Meta-analyses were performed using bivariate random-effects and univariate regression models for estimating summary performance measures across studies. Bivariate meta-regression was performed to explore the influence of testing method and study design. Subgroup and sensitivity analyses evaluated factors that may have led to heterogeneity. Based on 55 validation studies, the detection rate (DR) was significantly higher for retrospective studies, while the false positive rate (FPR) was significantly lower for prospective studies. Comparing the performance of NIPT methods for trisomies 21, 18, and 13 combined, the SNP method had a higher DR and lower FPR than other methods, significantly so for MPSS, though not for DANSR. The performance of the different methods in the 84 clinical experience studies was consistent with validation studies. Clinical positive predictive values of all NIPT methods improved over the last decade. We conclude that all NIPT methods are highly effective for fetal aneuploidy screening, with performance differences across methodologies.

## 1. Introduction

Non-invasive prenatal testing (NIPT) describes a family of tests that rely on the analysis of cell-free DNA fragments in the plasma of pregnant women to screen for fetuses affected by the common autosomal trisomies (trisomy 21, 18, and 13). Some NIPT laboratories also test for sex chromosome abnormalities (Turner syndrome, Klinefelter syndrome, XXX, XYY, and various more complex karyotypes), other autosomal aneuploidy, chromosome segmental imbalances (typically, >7 Mb), select microdeletion syndromes, Rhesus blood group typing, and some monogenic disorders [1].

Initial clinical validation studies were focused primarily on the prenatal identification of the common autosomal trisomies by detecting an overall quantitative difference in the proportion of cfDNA for those chromosomes where a copy number difference could exist [2,3,4]. The analysis requires a sufficiently high amount of circulating cfDNA derived from the conceptus such that fetal aneuploidy would be detectable, even in the presence of the larger quantities of cfDNA of maternal origin. Ensuring a sufficient “fetal fraction” (the proportion of cfDNA derived from placental trophoblasts) was therefore a key factor in the development of successful testing. Generally, adequate fetal fraction is achievable for testing after 9 or 10 weeks gestational age [5].

The methods used for cfDNA targeting, amplification, measurement, and data analysis differ considerably between laboratories. Massively parallel shotgun sequencing (MPSS)-based NIPT involves amplification of cfDNA across the whole genome with quantitative measurement of the DNA fragments present [6,7]. The bioinformatics used in this methodology involves a normalization of the observed sequence frequency based on expected amplification and measurement biases and comparison of the proportion of cfDNA from target chromosomes with that seen for control chromosomes. A targeted enrichment approach, referred to as digital analysis of selected regions (DANSR), involves the targeted amplification of key chromosomal regions involved in the most clinically important chromosome imbalances [3]. This approach allows reduced cost, deeper sequencing, and/or the use of alternative measurement technologies such as chromosome microarrays for the detection of imbalances. Similar to MPSS, it involves a quantitative comparison of the cfDNA present. An additional technical approach involves the selective amplification and analysis of a panel of single nucleotide polymorphisms (SNPs) which are likely to differ between mother and fetus. Chromosomal imbalances are detected by comparing observed SNP data to a set of hypothetical SNP distributions representing euploid and aneuploid states across different fetal fractions [8,9]. This latter approach can substantially distinguish between maternal versus fetal imbalances and detect imbalances such as triploidy and uniparental disomy that do not have proportional copy number differences between the chromosomes. Other methods have also been developed with variable scope for the chromosome regions tested.

All methods have strengths and weaknesses, and none should be considered fully diagnostic. Reasons for false-positive and false-negative results can include mosaicism, which may be confined to trophoblasts or the fetus, maternal chromosome abnormalities including both constitutional imbalances or somatically acquired abnormalities (including those associated with maternal cancer), and fetal death of a co-twin (“vanished twin”; often associated with an abnormal karyotype) [10]. Some of these factors are limitations for all NIPT methods (for example, confined placental mosaicism), while the likelihood of other false results is expected to be dependent on the technology used. Low fetal fraction (FF) is a potential source of error, and therefore, clinical laboratories typically establish a minimum FF cut-off below which the results are considered unreliable [11]. Low FF can also be associated with trisomy 13, 18, and triploidy-affected pregnancies where the placentas are usually small. Consequently, there is a reduction of fetal cfDNA in maternal plasma in those clinical situations [12].

Numerous studies have documented clinical validation and early clinical experience with NIPT. Prior meta-analyses have primarily considered the performance of all methods together and concluded that cfDNA provides an effective screening approach for fetal aneuploidy [13,14,15,16]. In this paper, we re-evaluated clinical validation studies distinguishing between testing methodologies and also between prospective versus retrospective study designs. Separately, we considered clinical experience studies that document actual patient care with NIPT and where confirmation of results was limited. We focused on NIPT for trisomies 21, 18, and 13 and monosomy X, where there has been the most extensive reporting of validation and experience.

## 2. Methods

**Figure 1:**
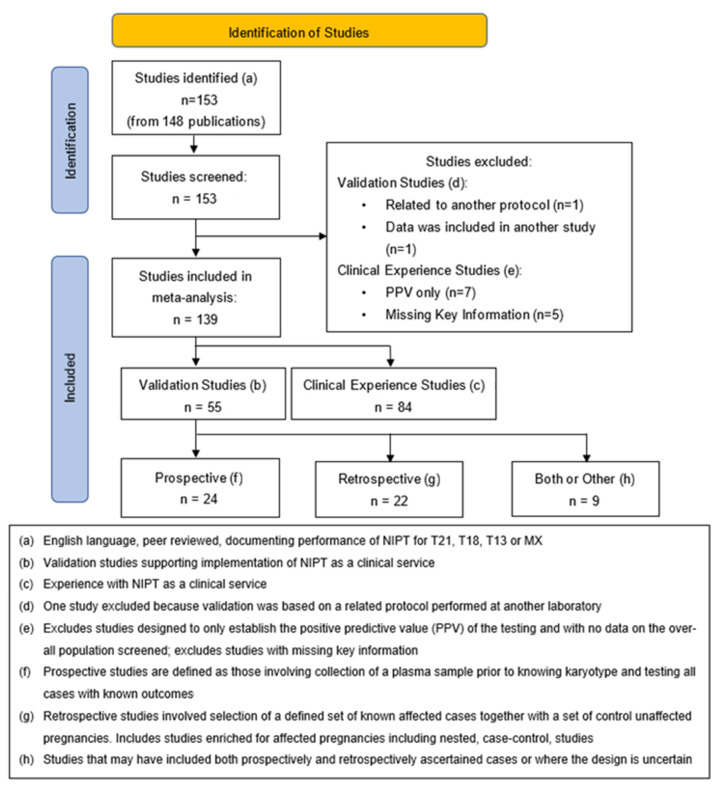
Flow diagram for the classification of the data extracted from the literature.

PubMed was searched for English language references using combinations of “prenatal”, “non-invasive”, “cell-free DNA”, “NIPT”, “NIPS”, “screening”, “trisomy”, and “aneuploidy”, for publications between January 2011 and January 2022. Relevant studies were reviewed for cross-references to additional studies that documented performance of cfDNA screening for trisomies 21, 18, 13, and monosomy X (MX). The approach to the meta-analysis was consistent with PRISMA guidelines with initial data extraction by one author (PB), independent review by a second author (ZD), and with resolution of discordances through joint review [17]. Figure 1 presents a flow diagram for the classification of the data extracted from the literature. The total number of references initially extracted was not recorded because there were multiple overlapping searches with early exclusion of ineligible studies.

Because of potential ascertainment biases (see below), studies were subclassified as either “validation” or “clinical experience”. “Validation” was defined as a study in which a set of maternal plasma samples, drawn at varying first- or second-trimester gestational ages, were tested for the presence or absence of fetal chromosome abnormality and where the actual aneuploidy status of the pregnancy (“truth”) was known for all samples included in the analysis. NIPT validation studies were typically conducted prior to the formal use in clinical practice and the test results were not used clinically. “Clinical experience” studies were defined as actual experience of a laboratory service that was routinely providing cfDNA screening for the purposes of patient management. Clinical experience studies typically involved the laboratory requesting follow-up data from ordering physicians for cases with high-risk but not low-risk NIPT results. Consequently, knowledge of outcomes was incomplete. For both validation and clinical experience studies, results of cytogenetic or cytogenomic analyses of amniotic fluid cells or chorionic villus sampling (CVS) or the occurrence of a normal livebirth or abnormal birth with the expected phenotype were considered evidence of truth. Without confirmatory genetic testing, the presence of either abnormal ultrasound findings or a spontaneous abortion was not considered sufficient evidence for aneuploidy. Because most NIPT were not designed to detect mosaicism, we excluded cases where a mosaic karyotype was detected in follow-up genetic testing. Where separately identified, twins or higher multiples were also excluded. Samples not yielding a result were also excluded.

Meta-analyses of test performance including all eligible studies were performed separately for “validation” and “clinical experience” studies. The outcome measures assessed and statistical methods applied were chosen based on what was most appropriate for each study subclassification as described below. We conducted statistical analyses using R, a language and environment for statistical computing and graphics. The R package mada was used for diagnostic meta-analysis implementing the approach of Reitsma et al., 2005 [18].

### 2.1. Validation Studies

Validation studies were further sub-classified as either “retrospective” or “prospective.” Retrospective was defined as a study where a set of maternal plasma specimens were collected and frozen, and samples were only analyzed if truth was known (mostly from CVS or amniocentesis samples) and sample truth status conformed to inclusion criteria in the study design. Case-control studies were considered to be retrospective for the purpose of this analysis. Prospective studies were based on a set of cases where cfDNA screening and the decision to include samples in the analysis was carried out prior to the knowledge of truth and where efforts were made to gather outcome on all tested cases. In some studies, methods were insufficiently documented to determine whether the design was prospective or retrospective.

For the validation studies, the outcome measures to assess test performance were prevalence (Prev), detection rate (DR, sensitivity), false positive rate (FPR, 1-specificity), diagnostic odds ratio (DOR), and positive predictive value (PPV). These outcome measures were based on the formulae below. Screen positive, affected cases were considered true positives (TP); screen negative, affected cases were considered false negatives (FN); screen positive, unaffected cases were considered false positives (FP); screen negative, unaffected cases were considered true negatives (TN).

$$Prev\;=\;\frac{TP\;+\;FN}{Total\;number\;of\;women\;with\;a\;screening\;result}\;DR\;=\;\frac{TP}{TP\;+\;FN}\;FPR\;=\;\frac{FP}{TN\;+\;FP}\;DOR\;=\;\frac{sensitivity\;\times \;specificity}{\left(1\;-\;sensitivity\right)\;\times \;\left(1\;-\;specificity\right)}\;=\;\frac{LR_{+}}{LR_{-}}\;=\;\frac{TP\;\times \;TN}{FP\;\times \;FN}$$

The DOR is defined as the ratio of the likelihood of the test being positive for an affected case (LR_+_) relative to the likelihood of the test being negative for an unaffected case (LR_−_). A higher DOR is indicative of better test performance. A rationale for using this single measure is that it is independent of prevalence and includes information about both sensitivity and specificity. However, this independence means that it cannot distinguish between tests with high sensitivity and low specificity and tests with low sensitivity and high specificity. Therefore, it was important to also implement methods considering both performance measures simultaneously.

$$PPV\;=\;\frac{TP}{TP\;+\;FP}\;=\;\frac{sensitivity\;\times \;prevalence}{\left(sensitivity\;\times \;prevalence\right)\;+\;\left(\left(1\;-\;specificity\right)\;\times \;\left(1\;-\;prevalence\right)\right)}$$

Differences in PPV observed in different studies may be attributable to differences in the proportion of affected pregnancies included in the various studies. To adjust for this difference in prevalence across studies, we used a standard prevalence for each syndrome representative of first trimester rates for a US population (population prev) [19]. The rates used were 1/365 for T21, 1/1208 for T18, 1/3745 for T13, and 1/1291 for MX. A standardized PPV (stdPPV) using the population prevalence was then calculated for each syndrome and testing method with the formula:

$$stdPPV\;=\;\frac{\left(sensitivity\;\times \;population\;prev\right)}{\left(sensitivity\;\times \;population\;prev\right)\;+\;\left(\left(1\;-\;specificity\right)\times \left(1\;-\;population\;prev\right)\right)}$$

Study-level data for validation studies were stratified by study type (retrospective and prospective) and by the three main methods of testing (MPSS, DANSR, and SNP). Other test methods were excluded due to insufficient data. Categorical variables were summarized as the proportion of studies within each category. Continuous variables were summarized by the median (25%ile and 75%ile). Forest plots showed the sensitivity and specificity for each study by syndrome (Trisomy 21, Trisomy 18, Trisomy 13, and Monosomy X).

Pooled estimates of the observed test performance were calculated as a descriptive summary of the data across studies by syndrome, method of testing, and study type. However, simple pooling is a fixed-effect method that ignores both the characteristics of the individual studies being pooled and the dependence of binary measures of test performance on the particular threshold used to determine the outcome. As the threshold is varied across all possible values, a trade-off is induced between sensitivity (DR) and specificity (1 − FPR). To take this into account, we used a bivariate approach, as recommended by the Cochrane Diagnostic Test Accuracy Working Group, to estimate the test performance across studies. Bivariate random effects regression models were used to estimate average sensitivity or detection rate (DR) and specificity (1 − FPR) through a joint distribution, accounting for the correlation between the two performance measures. This random effects approach incorporates unexplained variability in test performance measures between studies. Because there was also variation due to sampling, as studies differed in size, the precision by which DR and FPR were estimated in each study was incorporated by giving higher weight to studies with more precise estimates [18]. Because the model requires non-zero cells, we added a continuity correction of 0.1 to each cell of a study where a zero was encountered.

In addition to fitting models for each syndrome and method of testing or study type separately, bivariate meta-regression was performed on all validation study data to explore the influence of method of testing and study type by including them as covariates in the model. Studies using the SNP-based method were matched with studies using other methods with respect to study-level characteristics including start year, country, and prevalence. As a sensitivity analysis, meta-regression was performed for matched studies, for the subgroup of studies that differed from SNP-based studies with respect to study-level characteristics and for the full set of studies. Results were compared. Covariates were added to the bivariate model to examine their effects on DR and FPR as well as adjust for potential effects while testing for differences in performance by method of testing and study type. No corrections were made for multiple comparisons.

Summary ROC plots (sROC) showed the observed pairs of sensitivity (DR) and FPRs for each study as well as summary estimates of DRs and FPRs from bivariate models for each method of testing or study type including the corresponding 95% confidence ellipse showing the region of confidence that describes the uncertainty of the estimates.

### 2.2. Clinical Experience Studies

Clinical experience studies included reports from reference laboratories, regional fetal screening programs, and individual maternal–fetal medicine programs offering NIPT testing. Testing may have included prior conventional screening, with NIPT offered to high-risk women (“secondary screening”), or it may have been offered to a general pregnancy population (“primary screening”). For all clinical experience studies, the outcome data was judged incomplete. In particular, it is not possible to make a reliable estimate of DR because a proportion of test negative affected pregnancies will not come to attention. Even in the situation where there is a follow-up of a high proportion of screen-negative livebirths, there can be missed affected cases because of the relatively high risk of post-screening spontaneous pregnancy loss of aneuploid pregnancies. For this reason, TN were not tabulated for clinical experience studies. FN are included where reported, but it is important to note that these were largely data offered by ordering physicians, rather than requested by laboratories. As such, it is expected to be an underestimate. Analyses that only include participants for whom the outcome was obtained may produce biased estimates of test performance. We calculated the following test performance measures based on the observed data as well as using methods to correct for potential bias due to missing outcome data.

A minimum estimate of prevalence (minPrev) was calculated, recognizing the limitation that some false negatives are under-ascertained.

$$minPrev\;=\;\frac{\left(TP\;+\;FN\right)/Proportion\;of\;high\;risk\;calls\;with\;outcome\;data\;}{Number\;of\;women\;with\;results\;}$$

An approximate estimate of FPR (estFPR) was calculated on the basis that positive cases with follow-up were reflective of all positive cases and essentially all test negative cases were unaffected (see discussion).

$$estFPR\;=\;\frac{FP/Proportion\;of\;high\;risk\;calls\;with\;outcome\;data\;}{\left(FP/Proportion\;of\;high\;risk\;calls\;with\;outcome\;data\right)\;+\;\left(number\;of\;low\;risk\;calls\right)}$$

The Observed PPV (obsPPV) was based only on cases with outcome data.

$$obsPPV\;=\;\frac{Number\;of\;test\;positive\;confirmed\;affected\;cases}{Number\;of\;test\;positive\;cases\;with\;outcome\;data}$$

To correct for verification bias when calculating these performance metrics, we implemented inverse probability weighting. The number of confirmed high-risk calls was inflated by the inverse probability of having confirmation under the assumption that confirmation is missing at random for high-risk calls and positive cases with follow-up were reflective of all positive cases. We did not consider tests where no results were obtained. Standard statistical methods for verification bias correction have been shown to be inadequate when there are few false negatives. Therefore, the Observed PPVs were not considered to provide a reliable overall estimate and also did not reflect differences in prevalence. To allow for these factors, we also calculated and compared the standardized PPVs that incorporated the rate of false negatives among low-risk calls from validation studies and also used prevalence rates for a US population drawn from Benn et al. (2015) [19]. Study follow-up was calculated as the percentage of high-risk calls that were confirmed.

## 3. Results

### 3.1. Validation Studies

#### 3.1.1. Overall Performance

We identified 55 eligible validation studies, of which 22 were retrospective, 24 prospective, and 9 were both or of unknown study design (Supplemental Table S1). Of these 55 studies, 29 used an MPSS methodology, 13 used DANSR, 6 used SNP, and 7 used other methods. Supplemental Figure S1 summarizes, using forest plots, the sensitivity and specificity of these studies. Additional summary tabulation of these studies, by the three main methods of testing, publication year, year of laboratory testing, laboratory country, population country, gestational age, maternal age, and major study groups included are summarized in Supplemental Tables S2 and S3. Studies across the three methods of testing were comparable with respect to study-level characteristics. There appear to be differences between studies grouped by method of testing for start year, population country, and prevalence. These characteristics were used for matching in sensitivity analyses. Raw study-level data can be found in Supplemental Table S4.

For all validation studies combined, the pooled DR for T21 was 99.44% (95% CI 99.06–99.67%), and the FPR was 0.07% (CI 0.05–0.09%) (Table 1). From a bivariate random effects regression model, the mean DR for T21 was 98.72% (CI 97.97, 99.19%), and the FPR was 0.12% (CI 0.07, 0.21%) (Table 2). The DRs for T18, T13, and MX were lower than that for T21, but FPRs were similar.

Supplemental Table S5 shows the mean DORs stratified by study design, whether the validation was prospective or retrospective in design. Table 3 summarizes the mean DORs from a univariate model for each syndrome by testing method. For T21, T18 and T13, the highest DOR values were achieved with the SNP-based NIPT. For MX, the highest DOR was seen for DANSR.

For all syndromes, standardized PPVs were much lower than pooled observed PPVs for all syndromes due to the higher prevalence seen across the validation studies. The standardized PPV for T21 was 79.78% (95% CI 78.21–81.34), for T18 50.22% (95% CI 46.84–53.6), for T13 28.94% (95% CI 24.2–33.68), and for MX 13.17% (95% CI 9.12–17.23). Standardized PPV was highest for the SNP method for all syndromes.

#### 3.1.2. Retrospective Versus Prospective

Table 4 summarizes pooled estimates for the performance of testing by syndrome and study type. From a bivariate model, the mean DR for retrospective studies for T21 was 99.14% (95% CI 98.16–99.60) and for prospective studies 98.02% (95% CI 96.37–98.93) (Table 5). The mean FPR for retrospective studies for T21 was 0.21% (95% CI 0.08–0.57) and for prospective studies 0.09% (95% CI 0.05–0.17). The DRs for T18, T13, and MX were lower, but FPRs were similar. In a model controlling for syndrome, both the mean DR and mean FPR for retrospective studies were significantly higher than that of prospective studies (*p* = 0.004 and 0.003, respectively). Cohort prevalences for all syndromes were 4–6 times higher in retrospective compared to prospective studies. The differences in DR and FPR between prospective and retrospective studies were no longer statistically significant when including prevalence as a covariate in the model.

#### 3.1.3. Method of Testing

For T21, the mean FPR for SNP was significantly lower than that of MPSS among all validation studies (*p* = 0.029) (Table 2). In a sensitivity analysis that separately considered studies similar to, or different from, SNP-based studies with respect to study-level characteristics, the mean FPR for SNP remained statistically significantly lower than that of MPSS (*p* = 0.01 and *p* < 0.001, respectively). For T18, when considering matched studies or all studies combined, there was no statistically significant difference between methods. For T13 and MX, there were no statistically significant differences between methods of testing. When evaluating test performance in a model for each syndrome separately, there were no statistically significant differences for DR between methods of testing and no statistically significant difference between DANSR and SNP.

**Figure 2:**
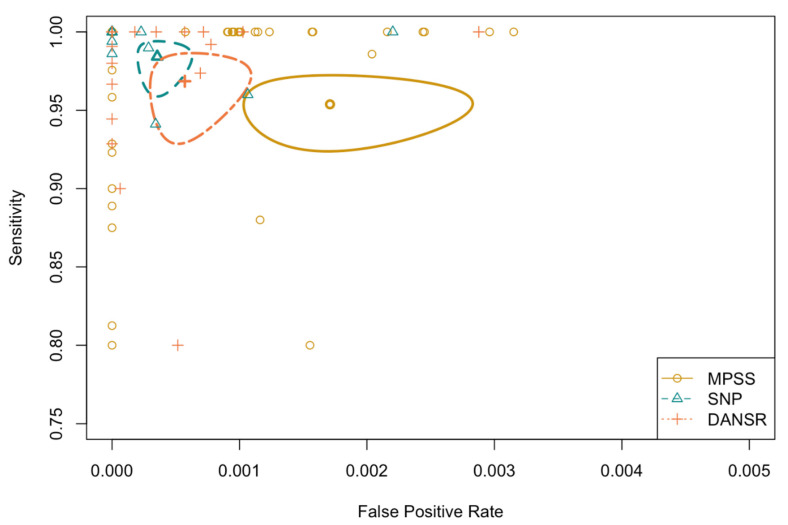
Summary Receiver Operating Curve for NIPT validation studies for common trisomies comparing MPSS, DANSR, and SNP.

To evaluate the relationship between DR/FPR and method of testing using data for all four syndromes combined, we fitted a bivariate model including syndrome and method of testing as covariates. The mean FPR for SNP was significantly lower than that of MPSS when controlling for syndrome among both matched studies (*p* = 0.046) and all studies combined (*p* = 0.008). When considering the three trisomies combined (no Monosomy X), the mean DR and FPR for SNP were significantly better than that of MPSS when controlling for syndrome among matched studies (*p* = 0.04 and *p* = 0.02, respectively) (Figure 2).

Figure 2 The summary ROC (sROC) plot shows the observed pairs of sensitivity and false positive rates (1-specificity) for each study as small symbols with summary estimates from bivariate models represented by the larger bolded symbols. Also shown are the corresponding 95% confidence ellipse showing the region of confidence that describes the uncertainty of the estimates for each method of testing. These plots typically show a curve estimated using a regression model meant to fit as close to the observed data as possible. The curve is estimated from 0 to 1 on both axes, but because the range of observed sensitivities and FPRs in our studies is limited, the model extends well beyond the range of the data to regions with limited or no data and therefore is not shown.

#### 3.1.4. Country

Because the studies conducted in China tended to have several differences from the other studies, namely, Chinese women tend to be of lower maternal weight, some early studies did not measure fetal fraction, some centers initially had compensation insurance for false negatives, testing was done mostly in the second trimester, and methods typically used lower sequencing depth, we performed a comparative analysis. The mean FPR for studies conducted in China was significantly lower than that of studies conducted in other countries (*p* = 0.01). To assess whether these findings influenced our results for differences between testing method, we performed a sensitivity analysis excluding studies conducted in China. The mean FPR for SNP remained significantly lower than that of MPSS when controlling for syndrome and excluding studies conducted in China (*p* = 0.001). In a model including the three trisomies (no Monosomy X), the mean FPR for SNP remained significantly lower than that of MPSS (*p* = 0.006), but the mean DR was no longer statistically significantly different (*p* = 0.07) when excluding Chinese studies.

Comparing studies conducted in European countries with those conducted in the US, the mean DR for US studies was significantly higher than that of European studies when controlling for syndrome and method of testing (*p* = 0.04).

### 3.2. Clinical Experience Studies

Overall Performance

A total of 84 eligible clinical experience studies were identified (Supplemental Table S1). Of these, 55 studies used MPSS, 12 DANSR, 12 SNP, and 5 used other technologies. Supplemental Table S6 summarizes the time of the study, laboratory country, population country, maternal age, and major indications for testing for those cases where the testing was based on MPSS, DANSR, or SNPs. Raw data from these studies are summarized in Supplemental Table S7. As summarized in Supplemental Table S8, the percentage of high-risk results where there was follow-up was highly variable.

Table 6 presents the estimated FPRs (estFPR), observed PPVs for confirmed cases (obsPPV), and standardized PPVs based on estimates of the population prevalence, as described in the Methods section. For confirmed cases for all syndromes, standardized PPVs were much lower than observed PPVs for confirmed cases for all syndromes. This difference can be explained by the higher prevalence seen across the clinical experience studies compared with the population rates used and the higher false negative rates used from the validation studies.

When results were stratified by study start year, the data were consistent with declining minimum prevalence estimates for each of the conditions (Table 7). Despite the declining prevalence estimates, PPV for confirmed cases increased with time for T21, T18, and T13 and remained approximately constant for MX.

We observed trends in test performance among the clinical experience studies consistent with those observed in the validation studies. Standardized PPVs for SNP trend higher than those for MPSS for all syndromes and FPRs for SNP trend lower than those for MPSS for all syndromes.

## 4. Discussion

In this study, we corroborate and update previous meta-analyses that showed that both validation studies and clinical experience studies demonstrate high efficacy of cell-free DNA in the prenatal screening of fetal trisomies 21, 18, 13, and monosomy X.

We show here that performance in validation studies depends on study design, with FPRs in prospective studies being significantly lower than in retrospective studies. Retrospective study design generally focused on ascertainment based on affected pregnancies identified through amniocentesis or CVS, i.e., unambiguous affected or unaffected pregnancies with mosaic cases, pregnancy losses, and unrelated abnormalities excluded. These studies are usually weighted to include sufficient affected pregnancies to allow robust estimation of detection rates. In prospective studies, there are variable policies with respect to the inclusion of cases with fetal loss, mosaicism, and other abnormality. Generally, prospective studies involve maternal plasma sample collection at the time of conventional maternal serum screening, and therefore, these studies are more representative of an average risk population, although inclusions may be weighted towards women with higher risks who undergo additional testing. The major distinction between the two designs appears to be prevalence. Our data show that after adjusting for prevalence, differences in DR and FPR between prospective and retrospective studies were no longer statistically significant.

This observation that testing performance is related to prevalence runs counter to classical views in screening where DR and FPR are considered intrinsic to the test and independent of the population screened. However, NIPT differs from conventional screening because many false positives have a biological, non-technical basis. For example, fetal or placental mosaicism can explain some false-positive results. Mosaicism can arise through a primary meiotic error (with correction to disomy via trisomy rescue), and the frequency of these cases can be expected to be maternal age dependent. Similarly, false positives due to a vanished twin, maternal cancer, somatic X-chromosome loss, and other maternal health conditions are also anticipated to be dependent on maternal age. A lower FPR and better than expected PPV in younger women compared to older women has been described [20]. An association between test performance and prevalence has been shown in other screening test settings [21,22]. Therefore, it is important to consider prevalence when assessing differences in study population or design.

We also found that the performance of the three main clinically available methods differed according to chromosome, with the SNP and DANSR methods trending towards better performance as compared to MPSS, although the differences were often not statistically significant. When considering the three trisomies combined and when controlling for syndrome among matched studies, the SNP method showed significantly better performance than MPSS. The trends were most clear when considering the diagnostic odds ratio, a measure that combines sensitivity and specificity. The same trends were also seen in the clinical experience data, where prevalence-adjusted PPVs provided a composite assessment of performance (Table 4).

NIPT has received increasing acceptance in medical practice, and consistent with this, we observed a trend towards declining prevalence for all four aneuploidies in women receiving the testing from 2010 to 2019 as the proportion of high-risk women in the testing population decreased (Table 5). Although this might be expected to result in lower PPV, in practice, the observed PPVs remained approximately constant. This may, in part, be explained by our additional observation that test performance is not independent of prevalence. It is likely also that improvements in testing have occurred. We acknowledge the observed PPVs are subject to verification bias, the effect of which could lead to less accurate results.

For clinical experience studies, we evaluated observed PPV and estimated FPR under the assumption that test-positive cases with definitive diagnosis or pregnancy outcome information are representative of all test-positive cases. In our experience, this tendency is primarily driven by the fact that some practices often do not have the time to respond to requests for follow-up information. Additionally, although it has been strongly recommended that all women with positive NIPT results receive definitive follow-up CVS or amniocentesis, follow-up is generally incomplete, and it is possible that in some cases with high-risk NIPT results where major malformations were detected by ultrasound, women chose to terminate their pregnancy without prenatal diagnostic testing. Conversely, it is possible some women with positive NIPT results that would be expected to show major malformations by ultrasound exam (e.g., trisomy 13 and 18) may choose not to pursue invasive testing in the absence of ultrasound findings. Additional difficulties exist for MX where mosaicism, partial X-chromosome deletions or unbalanced rearrangements, and the associated variable presentation are confounders. While drawing conclusions from positive cases with incomplete follow-up may therefore seem tenuous, our analysis does indicate that the FPRs and prevalence-adjusted PPVs in clinical practice are, in fact, consistent with the validation studies.

A greater difficulty exists in assessing DR from clinical experience studies. First, although some studies did report follow-up on FN that was offered, the majority of clinical experience studies did not request follow-up on cases with a low-risk result. Additionally, a high proportion of false-negative pregnancies may result in spontaneous losses without coming to attention. Others could have resulted in live-borns but not have been reported to the physician who ordered the NIPT. Some laboratories have attempted to infer DR based on extrapolation of outcome data from positive tests [23,24]. However, given the entirely different patient management of positive versus negative cases and the lack of supporting data to validate their underlying assumptions, these estimates must be viewed as lacking an evidence base.

We have not considered samples that did not provide results, as very few of the studies reported outcome on such cases. It is known that most of these are attributable to low fetal fraction, which is dependent on maternal weight and gestational age. Furthermore, there are no absolute standards for the measurement of fetal fraction or the threshold at which testing can be considered reliable. With low fetal fraction, there is a trade-off between successful testing that provides results and accuracy, both of which need to be considered when comparing test methodologies. Various strategies have been proposed to deal with low fetal fraction, including reflexively resequencing at a higher depth of read, re-assessing risk based on fetal fraction as a biomarker, use of artificial intelligence, use of alternative screening, or proceeding directly to diagnostic testing. As cfDNA testing is refined, the challenge posed by a low fetal fraction is being minimized.

Our review and analysis have limitations. We restricted our search to English language references, and some studies did not contain sufficient information necessary for inclusion. We were restricted to the study-level characteristics that we were able to extract, which limited our ability to assess and account for heterogeneity in the analyses. Furthermore, we did not assess the quality of individual studies. Due to the challenges with ascertainment bias in the clinical experience studies previously described, we were required to make assumptions and adjustments to calculate certain test performance measures. The bivariate random-effects regression model required a correction to be added to zero cells; this is known to lead to an underestimate of the performance in cases where the FNR and FPR are considerably lower than the correction factor, though this is not expected to affect overall trends. We used the method to account for various sources of bias and acknowledge our attempts may be inadequate. We did not evaluate the performance of NIPT for microdeletion syndromes and other chromosome imbalances. These newer additional areas of testing are not offered by all laboratories or may be limited to specific risk groups. Women with high prior risks due to abnormal ultrasound findings, family history, or maternal serum screening tests often proceed directly to cytogenetic and microarray diagnostic testing through CVS or amniocentesis. Exclusion of high-risk populations will lower the observed screening test PPVs.

In summary, we have shown that prospective validation studies demonstrate the excellent performance of NIPT for trisomies 21, 18, 13, and monosomy X and that methodological performance differences exist. The available data from clinical experience studies show that the performance of NIPT in clinical care is consistent with FPRs and PPVs obtained in clinical validation studies.

## Figures and Tables

**Table 1:** Validation studies: pooled estimates by syndrome.

Syndrome	Method	No. Called	Prevalence	Sensitivity	Specificity	PPV	Std PPV
T21	All	74697	3.35 (3.22, 3.48)	99.44 (99.06, 99.67)	99.93 (99.91, 99.95)	98.03 (97.41, 98.50)	79.78 (78.21, 81.34)
	MPSS	24308	5.14 (4.87, 5.42)	99.68 (99.18, 99.88)	99.88 (99.83, 99.92)	97.88 (96.93, 98.54)	70.05 (67.53, 72.57)
	DANSR	26208	1.91 (1.75, 2.08)	99.20 (97.96, 99.69)	99.96 (99.92, 99.98)	97.83 (96.16, 98.78)	86.43 (83.45, 89.41)
	SNP	19822	2.12 (1.93, 2.33)	99.29 (97.92, 99.76)	99.97 (99.94, 99.99)	98.82 (97.26, 99.49)	91.37 (88.69, 94.05)
T18	All	72847	1.11 (1.04, 1.19)	96.43 (94.92, 97.50)	99.92 (99.90, 99.94)	93.21 (91.31, 94.73)	50.22 (46.84, 53.60)
	MPSS	23508	1.77 (1.61, 1.95)	95.68 (93.28, 97.25)	99.89 (99.84, 99.93)	94.10 (91.44, 95.97)	42.27 (37.57, 46.97)
	DANSR	25619	0.67 (0.57, 0.77)	97.66 (94.14, 99.09)	99.96 (99.93, 99.98)	94.35 (89.91, 96.90)	67.27 (60.35, 74.18)
	SNP	19823	0.53 (0.44, 0.65)	98.11 (93.38, 99.48)	99.96 (99.93, 99.98)	93.69 (87.55, 96.91)	69.60 (61.04, 78.16)
T13	All	64640	0.50 (0.44, 0.55)	97.19 (94.74, 98.51)	99.94 (99.91, 99.95)	88.35 (84.58, 91.30)	28.94 (24.20, 33.68)
	MPSS	23470	0.72 (0.62, 0.84)	96.47 (92.51, 98.37)	99.88 (99.83, 99.92)	85.42 (79.73, 89.71)	17.66 (12.26, 23.05)
	DANSR	17629	0.29 (0.22, 0.38)	96.08 (86.78, 98.92)	99.97 (99.93, 99.99)	90.74 (80.09, 95.98)	47.43 (34.11, 60.75)
	SNP	19823	0.30 (0.24, 0.39)	100 (93.98, 100)	99.97 (99.94, 99.99)	92.31 (83.22, 96.67)	51.36 (39.21, 63.51)
MX	All	15079	1.34 (1.17, 1.54)	96.04 (92.38, 97.98)	99.51 (99.38, 99.61)	72.66 (67.02, 77.66)	13.17 (9.12, 17.23)
	MPSS	10552	1.05 (0.87, 1.27)	94.59 (88.71, 97.50)	99.40 (99.23, 99.53)	62.50 (54.98, 69.46)	10.84 (6.14, 15.54)
	DANSR	2481	2.78 (2.20, 3.50)	100 (94.73, 100)	99.63 (99.29, 99.80)	88.46 (79.50, 93.81)	17.20 (8.83, 25.58)
	SNP	2046	1.08 (0.71, 1.62)	90.91 (72.19, 97.47)	99.95 (99.72, 100)	95.24 (77.33, 99.76)	58.79 (37.73, 79.84)
T21, T13, T18	All	212184	1.71 (1.66, 1.77)	98.57 (98.13, 98.91)	99.93 (99.92, 99.94)	96.03 (95.35, 96.61)	---
	MPSS	71286	2.58 (2.46, 2.69)	98.47 (97.80, 98.94)	99.88 (99.86, 99.91)	95.76 (94.76, 96.58)	---
	DANSR	69456	1.04 (0.97, 1.12)	98.61 (97.47, 99.25)	99.96 (99.94, 99.97)	96.48 (94.89, 97.58)	---
	SNP	59468	0.99 (0.91, 1.07)	99.15 (98.02, 99.64)	99.97 (99.95, 99.98)	97.16 (95.49, 98.22)	---
All	All	227263	1.69 (1.63, 1.74)	98.43 (97.99, 98.78)	99.90 (99.89, 99.91)	94.47 (93.71, 95.13)	---
	MPSS	81838	2.38 (2.28, 2.49)	98.25 (97.57, 98.75)	99.82 (99.79, 99.85)	93.04 (91.86, 94.07)	---
	DANSR	71937	1.10 (1.03, 1.18)	98.74 (97.69, 99.31)	99.95 (99.93, 99.96)	95.71 (94.09, 96.90)	---
	SNP	61514	0.99 (0.91, 1.07)	98.85 (97.64, 99.44)	99.97 (99.95, 99.98)	97.09 (95.45, 98.15)	---

**Table 2:** Validation studies: summary estimates for Sensitivity (Sens) and Specificity (Spec) from bivariate model, by syndrome and method.

Syndrome	Method	No. Studies	Mean Sens (95% CI)	Mean Spec (95% CI)
T21	All	48	98.72 (97.97, 99.19)	99.88 (99.79, 99.93)
	MPSS	26	99.05 (97.95, 99.56)	99.82 (99.63, 99.91)
	DANSR	10	98.15 (94.97, 99.33)	99.95 (99.91, 99.97)
	SNP	5	99.07 (97.34, 99.68)	99.97 (99.93, 99.99)
T18	All	43	93.54 (90.99, 95.40)	99.86 (99.77, 99.92)
	MPSS	24	92.28 (88.28, 94.99)	99.83 (99.66, 99.91)
	DANSR	9	96.76 (91.70, 98.78)	99.93 (99.80, 99.98)
	SNP	5	96.25 (87.26, 98.97)	99.96 (99.92, 99.98)
T13	All	40	91.87 (86.13, 95.37)	99.90 (99.83, 99.95)
	MPSS	23	90.90 (82.39, 95.53)	99.86 (99.69, 99.94)
	DANSR	7	87.65 (64.99, 96.44)	99.96 (99.91, 99.98)
	SNP	6	98.77 (86.17, 99.90)	99.95 (99.76, 99.99)
MX	All	21	90.90 (83.09, 95.31)	99.70 (99.29, 99.88)
	MPSS	13	90.83 (81.24, 95.77)	99.70 (99.04, 99.90)
	DANSR	4	98.92 (78.66, 99.96)	99.45 (97.35, 99.89)
	SNP	4	85.04 (57.03, 96.05)	99.92 (99.56, 99.99)

**Table 3:** Validation studies: summary estimates for Diagnostic Odds Ratio (DOR) from univariate model, by syndrome and method.

Syndrome	Method	Mean DOR	(95% CI)
T21	All	35,173	(22,313, 55,444)
	MPSS	28,585	(15,104, 54,097)
	DANSR	61,486	(22,113, 170,963)
	SNP	108,829	(30,768, 384,936)
T18	All	7,212	(4395, 11,833)
	MPSS	5,195	(2655, 10,165)
	DANSR	18,403	(6755, 50,135)
	SNP	20,455	(6053, 69125)
T13	All	6,798	(3959, 11,673)
	MPSS	4,915	(2415, 10,004)
	DANSR	9,197	(2659, 31,807)
	SNP	14,206	(3215, 62,775)
MX	All	2,177	(1065, 4451)
	MPSS	1,826	(650, 5135)
	DANSR	3,898	(761, 19,976)
	SNP	3,262	(655, 16,256)

**Table 4:** Validation studies: pooled estimates by syndrome and study type.

Syndrome	Study Type	No. Called	Prevalence	Sensitivity	Specificity	PPV	Std PPV
T21	Retrospective	11847	8.92 (8.42, 9.45)	99.53 (98.9, 99.8)	99.88 (99.79, 99.93)	98.78 (97.92, 99.29)	69.41 (66.65, 72.18)
	Prospective	57892	1.68 (1.58, 1.79)	99.18 (98.38, 99.58)	99.94 (99.92, 99.96)	96.69 (95.38, 97.63)	82.45 (80.09, 84.82)
T18	Retrospective	11181	2.77 (2.48, 3.09)	98.39 (96.28, 99.31)	99.88 (99.79, 99.93)	95.91 (93.13, 97.60)	40.42 (35.03, 45.82)
	Prospective	57049	0.60 (0.54, 0.67)	95.63 (92.91, 97.33)	99.95 (99.93, 99.96)	91.88 (88.58, 94.28)	60.77 (55.71, 65.84)
T13	Retrospective	10737	1.23 (1.04, 1.46)	95.45 (90.44, 97.90)	99.82 (99.72, 99.89)	86.90 (80.44, 91.45)	12.46 (7.08, 17.83)
	Prospective	49321	0.22 (0.18, 0.27)	97.25 (92.22, 99.06)	99.97 (99.94, 99.98)	86.18 (78.98, 91.19)	42.92 (34.17, 51.67)
MX	Retrospective	5499	2.38 (2.01, 2.82)	96.18 (91.38, 98.36)	98.99 (98.69, 99.23)	70.00 (62.94, 76.22)	6.90 (3.20, 10.60)
	Prospective	6880	0.39 (0.27, 0.57)	88.89 (71.94, 96.15)	99.78 (99.64, 99.87)	61.54 (45.90, 75.11)	23.94 (10.55, 37.34)

**Table 5:** Validation studies: summary estimates for Sensitivity (Sens) and Specificity (Spec) from bivariate model, by syndrome and study type.

Syndrome	Type	N Studies	Mean Sens (95% CI)	Mean Spec (95% CI)
T21	Retrospective	16	99.14 (98.16, 99.60)	99.79 (99.43, 99.92)
	Prospective	24	98.02 (96.37, 98.93)	99.91 (99.83, 99.95)
T18	Retrospective	13	96.50 (92.30, 98.45)	99.83 (99.65, 99.92)
	Prospective	23	92.81 (88.66, 95.51)	99.91 (99.82, 99.95)
T13	Retrospective	12	88.35 (76.60, 94.61)	99.90 (99.62, 99.97)
	Prospective	21	93.17 (84.53, 97.15)	99.92 (99.86, 99.96)
MX	Retrospective	9	93.84 (86.14, 97.4)	99.55 (97.87, 99.91)
	Prospective	8	76.11 (49.09, 91.32)	99.67 (99.46, 99.80)

**Table 6:** Clinical experience studies: pooled estimates by syndrome and method of testing.

Syndrome	Method	FPR	Min Prevalence	PPV for Confirmed Cases	Std PPV
T21	All	0.050 (0.047, 0.053)	0.68 (0.67, 0.69)	92.48 (91.88, 93.05)	84.34 (83.80, 84.88)
	MPSS	0.056 (0.052, 0.060)	0.60 (0.59, 0.61)	91.82 (91.10, 92.48)	82.72 (81.91, 83.53)
	DANSR	0.026 (0.012, 0.056)	1.16 (1.03, 1.30)	97.41 (94.08, 98.89)	91.40 (88.11, 94.69)
	SNP	0.042 (0.039, 0.047)	0.74 (0.73, 0.76)	94.39 (93.03, 95.50)	86.37 (85.65, 87.10)
T18	All	0.040 (0.038, 0.043)	0.18 (0.18, 0.19)	78.47 (76.80, 80.06)	62.73 (61.42, 64.03)
	MPSS	0.058 (0.054, 0.062)	0.16 (0.16, 0.17)	75.37 (73.39, 77.24)	49.04 (47.18, 50.90)
	DANSR	0.039 (0.020, 0.074)	0.24 (0.19, 0.31)	79.55 (65.50, 88.85)	66.58 (54.84, 78.32)
	SNP	0.019 (0.016, 0.021)	0.20 (0.19, 0.21)	91.77 (88.72, 94.05)	80.81 (79.22, 82.39)
T13	All	0.050 (0.048, 0.053)	0.06 (0.06, 0.07)	48.00 (45.15, 50.86)	30.20 (28.47, 31.94)
	MPSS	0.059 (0.055, 0.063)	0.05 (0.04, 0.05)	43.66 (40.50, 46.86)	22.43 (20.17, 24.69)
	DANSR	0.083 (0.053, 0.129)	0.10 (0.07, 0.15)	54.17 (35.07, 72.11)	22.16 (8.95, 35.36)
	SNP	0.040 (0.036, 0.044)	0.08 (0.08, 0.09)	64.13 (56.98, 70.71)	40.23 (37.58, 42.89)
MX	All	0.095 (0.091, 0.100)	0.10 (0.10, 0.11)	31.05 (28.87, 33.33)	34.84 (33.37, 36.31)
	MPSS	0.161 (0.153, 0.169)	0.05 (0.05, 0.06)	25.92 (23.70, 28.26)	18.68 (16.90, 20.46)
	DANSR	0.072 (0.055, 0.094)	0.04 (0.03, 0.05)	31.43 (21.76, 43.03)	51.93 (40.91, 62.94)
	SNP	0.045 (0.042, 0.050)	0.15 (0.14, 0.16)	74.70 (67.58, 80.70)	50.71 (48.58, 52.83)
T21, T13, T18	All	0.047 (0.045, 0.048)	0.31 (0.30, 0.31)	84.89 (84.22, 85.53)	---
	MPSS	0.057 (0.055, 0.060)	0.27 (0.27, 0.28)	83.25 (82.46, 84.01)	---
	DANSR	0.049 (0.035, 0.068)	0.51 (0.46, 0.56)	90.42 (86.24, 93.43)	---
	SNP	0.034 (0.032, 0.036)	0.34 (0.33, 0.35)	90.95 (89.59, 92.15)	---
All	All	0.057 (0.056, 0.059)	0.26 (0.26, 0.27)	78.07 (77.35, 78.77)	---
	MPSS	0.076 (0.074, 0.079)	0.23 (0.23, 0.23)	75.39 (74.54, 76.21)	---
	DANSR	0.061 (0.049, 0.075)	0.27 (0.24, 0.30)	77.95 (73.17, 82.08)	---
	SNP	0.037 (0.035, 0.038)	0.29 (0.29, 0.30)	89.67 (88.29, 90.90)	---

**Table 7:** Clinical experience studies: pooled estimates by syndrome and study start year.

Syndrome	Start Year	Tech	Min Prevalence	PPV for Confirmed Cases
T21	2010–2013	All	0.84 (0.81, 0.86)	90.95 (89.82, 91.96)
	2014–2016	All	0.64 (0.63, 0.66)	92.39 (91.43, 93.25)
	2017–2019	All	0.45 (0.42, 0.47)	94.08 (92.61, 95.27)
T18	2010–2013	All	0.21 (0.19, 0.22)	70.00 (66.82, 73.00)
	2014–2016	All	0.18 (0.17, 0.18)	82.39 (79.99, 84.56)
	2017–2019	All	0.13 (0.12, 0.14)	80.21 (75.91, 83.91)
T13	2010–2013	All	0.05 (0.05, 0.06)	40.27 (35.40, 45.34)
	2014–2016	All	0.06 (0.06, 0.07)	44.64 (40.14, 49.22)
	2017–2019	All	0.05 (0.04, 0.06)	56.65 (50.23, 62.86)
MX	2010–2013	All	0.10 (0.09, 0.12)	30.99 (26.72, 35.61)
	2014–2016	All	0.13 (0.12, 0.13)	30.47 (27.19, 33.95)
	2017–2019	All	0.04 (0.03, 0.04)	31.01 (27.02, 35.32)

## Data Availability

All the data is provided in the manuscript and supplementary materials.

## Supplementary Materials

The following supporting information can be downloaded at: https://www.mdpi.com/article/10.3390/jcm11164760/s1, Table S1: Validation and Clinical Experience Papers Included and Excluded from Analysis with References. Table S2: Validation Study Characteristics Stratified by NIPT Method. Table S3: Validation Study Characteristics Stratified by Study Design. Table S4: Excel document with data extracted from manuscripts of all Validation studies. (‘All Validation’ tab). Table S5: Mean Diagnostic Odds Ratios Stratified by Study Design. Table S6: Summary of Clinical Experience Study Characteristics by NIPT Methodology. Table S7: Excel document with data extracted from manuscripts of all Clinical Experience studies. (‘All Clinical Experience’ tab). Table S8: Number and Percentage of High-Risk Results Where There was Follow-up. Figure S1: Forest Plots of all Data. References [25,26,27,28,29,30,31,32,33,34,35,36,37,38,39,40,41,42,43,44,45,46,47,48,49,50,51,52,53,54,55,56,57,58,59,60,61,62,63,64,65,66,67,68,69,70,71,72,73,74,75,76,77,78,79,80,81,82,83,84,85,86,87,88,89,90,91,92,93,94,95,96,97,98,99,100,101,102,103,104,105,106,107,108,109,110,111,112,113,114,115,116,117,118,119,120,121,122,123,124,125,126,127,128,129,130,131,132,133,134,135,136,137,138,139,140,141,142,143,144,145,146,147,148,149,150,151,152,153,154,155,156,157,158,159,160,161,162,163,164,165,166] are cited in the supplementary materials.
