# Supplementary material for: A Critical Evaluation of Validation and Clinical Experience Studies in Non-Invasive Prenatal Testing for Trisomies 21, 18, and 13 and Monosomy X
Source: J Clin Med. 2022 Aug 15;11(16):4760. doi: 10.3390/jcm11164760 (PMC9410356; doi:10.3390/jcm11164760)
Supplement: Supplementary file 1 [file jcm-11-04760-s001.zip › Demko et al. NIPT Meta-analysis Supplemental Material _June 23_Final.pdf]

Journal of
Clinical Medicine

MDPI

Supplemental Table S1. Validation and Clinical Experience Papers Included and Excluded from Analysis with References.

Validation papers included in the analysis:

Alberti [25]	Ehrich [35]	Koumbaris [46]	Norton [57]	Porreco [67]
Arigul [26]	El Khattabi [36]*	Kypri [47]*	Norton [58]	Ryan [68]
Ashoor [27]	Ericsson [37]	Langlois [48]	Palomaki [2]	Sehnert [69]
Ashoor [28]	Flöck [38]	Lau [49]	Palomaki [59]	Shaw [70]
Benachi [29]	Guex [39]	Lee [50]	Papageorghiou [60]	Song [71]
Bianchi [4]	Gormus [40]	Liang [51]	Pergament [9]	Song [72]
Bianchi [30]	Hall [41]	Mazloom [52]	Persico [62]	Stokowski [73]
Chen [31]	Hu [42]	Miltoft [53]	Pertile [63]	Stumm [74]
Chiu [32]	Jeon [43]	Nicolaides [54]	Pooh [64]	Tsaliki [75]
Dahl [33]	Jiang [44]	Nicolaides [55]	Poon [65]	Tynan [76]
Dar [34]	Ke [45]	Nicolaides [56]	Porreco [66]	Verweij [77]

* Study contained a validation and clinical experience component and were included in both.

Validation papers not included in the analysis:

Reference	Reason
Persico [61]	Data was reported in Persico [62]
Strom§ [138]	Study was related to another protocol

§ Study contained a validation and clinical experience component but was excluded from validation analysis.

Clinical experience papers included in the analysis:

Alyafee [78]	El Khattabi [36]*	Jackson [103]	Luo [116]	Qi [130]	Wang [143]
Bajka [79]	Fairbrother [91]	Kagan [104]	Luo [117]	Quezada [131]	Wang [144]
Beamon [80]	Fiorentino [92]	Koc [105]	Luthgens [118]	Radoi [132]	Wang [145]
Bianchi [81]	Futch [93]	Korostelev [106]	Manotaya [119]	Sainz [133]	Willems [146]
Borth [82]	Garshasbi [94]	Kypri [47]*	Margiotti [120]	Santamaria [134]	Xu [147]
Bu [83]	Ge [95]	La Verde [107]	Mesoraca [121]	Sasaki [135]	Xu [148]
Chen [84]	Gil [96]	Lai [108]	Noh [122]	Serapinas [136]	Xue [149]^
Comas [85]^	Gil [97]	Lau [109]	Oepkes [123]	Song [137]	Yang [150]
Dar [86]	Guy [98]	Liang [110]	Oneda [124]	Strom [138]	Zhang [151]
Dai [87]	Guy [99]	Liu [111]	Palomaki [125]	Taneja [23]	Zheng [152]
Dan [88]	Hancock [24]	Lu [112]	Panchalee [126]	Togneri [139]	Zheng [153]
DiNonno [20]	Harasim [100]	Lu [113]	Pang [127]	van der Meij [140]	Zhu [154]
Du [89]	Hong [101]	Lu [114]	Pavanello [128]	Verma [141]	
Eiben [90]	Hu [102]	Lund [115]	Pescia [129]	Wan [142]	

* Study contained a validation and clinical experience component and were included in both.

^ Study includes data from different platforms and were managed separately in the analyses.

Clinical experience papers not included in the analysis:

Reference	Reason
Dougan [155]	Insufficient detail
Gug [156]	Insufficient detail
McCullough [157]	No TP/FP numbers
Meck [158]	Only positive tests evaluated
Saito [159]	Insufficient detail
Suzumori [160]	Discrepant numbers
Tekesin [161]	Only positive tests evaluated
Togneri [162]	Only positive tests evaluated
Valderramos [163]	Only positive tests evaluated
Van Den Bogaert [164]	Only positive tests evaluated
Wang [165]	Only positive tests evaluated
Wu [166]	Only positive tests evaluated

Supplementary References

2. Palomaki, G.E.; Kloza, E.M.; Lambert-Messerlian, G.M.; Haddow, J.E.; Neveux, L.M.; Ehrich, M.; van den Boom, D.; Bombard, A.T.; Deciu, C.; Grody, W.W. DNA sequencing of maternal plasma to detect Down syndrome: an international clinical validation study. *Genetics in Medicine* **2011**, *13*, 913-920.

4. Bianchi, D.W.; Platt, L.D.; Goldberg, J.D.; Abuhamad, A.Z.; Sehnert, A.J.; Rava, R.P. Genome-wide fetal aneuploidy detection by maternal plasma DNA sequencing. *Obstetrics & Gynecology* **2012**, *119*, 890-901.

9. Pergament, E.; Cuckle, H.; Zimmermann, B.; Banjevic, M.; Sigurjonsson, S.; Ryan, A.; Hall, M.P.; Dodd, M.; Lacroute, P.; Stosic, M. Single-nucleotide polymorphism–based noninvasive prenatal screening in a high-risk and low-risk cohort. *Obstetrics & Gynecology* **2014**, *124*, 210.

20. DiNonno, W.; Demko, Z.; Martin, K.; Billings, P.; Egbert, M.; Zneimer, S.; Keen-Kim, D.; Benn, P. Quality assurance of non-invasive prenatal screening (NIPS) for fetal aneuploidy using positive predictive values as outcome measures. *Journal of Clinical Medicine* **2019**, *8*, 1311.

23. Taneja, P.A.; Snyder, H.L.; de Feo, E.; Kruglyak, K.M.; Halks-Miller, M.; Curnow, K.J.; Bhatt, S. Noninvasive prenatal testing in the general obstetric population: clinical performance and counseling considerations in over 85 000 cases. *Prenatal Diagnosis* **2016**, *36*, 237-243, doi:10.1002/pd.4766.

24. Hancock, S.; Ben-Shachar, R.; Adusei, C.; Oyolu, C.B.; Evans, E.A.; Kang, H.P.; Haverty, C.; Muzzey, D. Clinical experience across the fetal-fraction spectrum of a non-invasive prenatal

screening approach with low test-failure rate. *Ultrasound in Obstetrics & Gynecology* **2020**, *56*, 422-430, doi:10.1002/uog.21904.

25. Alberti, A.; Salomon, L.; Le Lorc'h, M.; Couloux, A.; Bussieres, L.; Goupil, S.; Malan, V.; Pelletier, E.; Hyon, C.; Vialard, F. Non-invasive prenatal testing for trisomy 21 based on analysis of cell-free fetal DNA circulating in the maternal plasma. *Prenatal Diagnosis* **2015**, *35*, 471-476.
26. Arigul, T.; Suwannachairob, W.; Moungklom, N.; Praphanphoj, V. Sensitivity and specificity of MGC-NIPS for trisomy 13, trisomy 18, trisomy 21, and sex chromosome aneuploidy screening in 219 Thai pregnant women. *Genomics and Genetics* **2020**, *13*, 1-6.
27. Ashoor, G.; Syngelaki, A.; Wagner, M.; Birdir, C.; Nicolaides, K.H. Chromosome-selective sequencing of maternal plasma cell–free DNA for first-trimester detection of trisomy 21 and trisomy 18. *American Journal of Obstetrics and Gynecology* **2012**, *206*, 322. e321-322. e325.
28. Ashoor, G.; Syngelaki, A.; Wang, E.; Struble, C.; Oliphant, A.; Song, K.; Nicolaides, K.H. Trisomy 13 detection in the first trimester of pregnancy using a chromosome-selective cell-free DNA analysis method. *Ultrasound in Obstetrics & Gynecology* **2013**, *41*, 21-25.
29. Benachi, A.; Letourneau, A.; Kleinfinger, P.; Senat, M.-V.; Gautier, E.; Favre, R.; Bidat, L.; Houfflin-Debarge, V.; Bouyer, J.; Costa, J.-M. Cell-free DNA analysis in maternal plasma in cases of fetal abnormalities detected on ultrasound examination. *Obstetrics & Gynecology* **2015**, *125*, 1330-1337.
30. Bianchi, D.W.; Parker, R.L.; Wentworth, J.; Madankumar, R.; Saffer, C.; Das, A.F.; Craig, J.A.; Chudova, D.I.; Devers, P.L.; Jones, K.W. DNA sequencing versus standard prenatal aneuploidy screening. *New England Journal of Medicine* **2014**, *370*, 799-808.
31. Chen, E.Z.; Chiu, R.W.; Sun, H.; Akolekar, R.; Chan, K.A.; Leung, T.Y.; Jiang, P.; Zheng, Y.W.; Lun, F.M.; Chan, L.Y. Noninvasive prenatal diagnosis of fetal trisomy 18 and trisomy 13 by maternal plasma DNA sequencing. *PloS One* **2011**, *6*, e21791.
32. Chiu, R.W.; Akolekar, R.; Zheng, Y.W.; Leung, T.Y.; Sun, H.; Chan, K.A.; Lun, F.M.; Go, A.T.; Lau, E.T.; To, W.W. Non-invasive prenatal assessment of trisomy 21 by multiplexed maternal plasma DNA sequencing: large scale validity study. *BMJ* **2011**, *342*.
33. Dahl, F.; Ericsson, O.; Karlberg, O.; Karlsson, F.; Howell, M.; Persson, F.; Roos, F.; Stenberg, J.; Ahola, T.; Alftrén, I. Imaging single DNA molecules for high precision NIPT. *Scientific Reports* **2018**, *8*, 1-8.
34. Dar, P.; Jacobson, B.; MacPherson, C.; Egbert, M.; Malone, F.; Wapner, R.J.; Roman, A.S.; Khalil, A.; Faro, R.; Madankumar, R.; et al. Cell-free DNA screening for trisomies 21, 18 and 13 in pregnancies at low and high risk for aneuploidy with genetic confirmation. *American Journal of Obstetrics and Gynecology* **2022**.
35. Ehrich, M.; Deciu, C.; Zwiefelhofer, T.; Tynan, J.A.; Cagasan, L.; Tim, R.; Lu, V.; McCullough, R.; McCarthy, E.; Nygren, A.O. Noninvasive detection of fetal trisomy 21 by sequencing of DNA in maternal blood: a study in a clinical setting. *American Journal of Obstetrics and Gynecology* **2011**, *204*, 205. e201-205. e211.

36. El Khattabi, L.A.; Brun, S.; Gueguen, P.; Chatron, N.; Guichoux, E.; Schutz, S.; Nectoux, J.; Sorlin, A.; Quere, M.; Boudjarane, J. Performance of semiconductor sequencing platform for non-invasive prenatal genetic screening for fetal aneuploidy: results from a multicenter prospective cohort study in a clinical setting. *Ultrasound in Obstetrics & Gynecology* **2019**, *54*, 246-254.
37. Ericsson, O.; Ahola, T.; Dahl, F.; Karlsson, F.; Persson, F.; Karlberg, O.; Roos, F.; Alftrén, I.; Andersson, B.; Barkenäs, E. Clinical validation of a novel automated cell-free DNA screening assay for trisomies 21, 13, and 18 in maternal plasma. Prenatal Diagnosis 2019, 39, 1011-1015.
38. Flöck, A.; Tu, N.-C.; Rüland, A.; Holzgreve, W.; Gembruch, U.; Geipel, A. Non-invasive prenatal testing (NIPT): Europe's first multicenter post-market clinical follow-up study validating the quality in clinical routine. Archives of Gynecology and Obstetrics 2017, 296, 923-928.
39. Guex, N.; Iseli, C.; Syngelaki, A.; Deluen, C.; Pescia, G.; Nicolaides, K.; Xenarios, I.; Conrad, B.; Conrad, B. A robust 2nd generation genome-wide test for fetal aneuploidy based on shotgun sequencing cell-free DNA in maternal blood. Prenatal Diagnosis 2013.
40. Gormus, U.; Chaubey, A.; Shenoy, S.; Wong, Y.W.; Chan, L.Y.; Choo, B.P.; Oraha, L.; Gousseva, A.; Persson, F.; Prensky, L. Assessment and Clinical Utility of a Non-Next-Generation Sequencing-Based Non-Invasive Prenatal Testing Technology. Current Issues in Molecular Biology 2021, 43, 958-964.
41. Hall, M.P.; Hill, M.; Zimmermann, B.; Sigurjonsson, S.; Westemeyer, M.; Saucier, J.; Demko, Z.; Rabinowitz, M. Non-invasive prenatal detection of trisomy 13 using a single nucleotide polymorphism-and informatics-based approach. PloS One 2014, 9, e96677.
42. Hu, H.-J.; Lee, M.-Y.; Cho, D.-Y.; Oh, M.; Kwon, Y.-J.; Han, Y.-J.; Ryu, H.M.; Kim, Y.N.; Won, H.-S. Prospective clinical evaluation of Momguard non-invasive prenatal test in 1011 Korean high-risk pregnant women. Journal of Obstetrics and Gynaecology 2020, 40, 1090-1095.
43. Jeon, Y.J.; Zhou, Y.; Li, Y.; Guo, Q.; Chen, J.; Quan, S.; Zhang, A.; Zheng, H.; Zhu, X.; Lin, J. The feasibility study of non-invasive fetal trisomy 18 and 21 detection with semiconductor sequencing platform. PLoS One 2014, 9, e110240.
44. Jiang, F.; Ren, J.; Chen, F.; Zhou, Y.; Xie, J.; Dan, S.; Su, Y.; Xie, J.; Yin, B.; Su, W. Noninvasive Fetal Trisomy (NIFTY) test: an advanced noninvasive prenatal diagnosis methodology for fetal autosomal and sex chromosomal aneuploidies. BMC Medical Genomics 2012, 5, 1-11.
45. Ke, W.-L.; Zhao, W.-H.; Wang, X.-Y. Detection of fetal cell-free DNA in maternal plasma for Down syndrome, Edward syndrome and Patau syndrome of high risk fetus. International Journal of Clinical and Experimental Medicine 2015, 8, 9525.
46. Koumbaris, G.; Kypri, E.; Tsangaras, K.; Achilleos, A.; Mina, P.; Neofytou, M.; Velissariou, V.; Christopoulou, G.; Kallikas, I.; González-Liñán, A. Cell-free DNA analysis of targeted genomic regions in maternal plasma for non-invasive prenatal testing of trisomy 21, trisomy 18, trisomy 13, and fetal sex. Clinical Chemistry 2016, 62, 848-855.

47. Kypri, E.; Ioannides, M.; Touvana, E.; Neophytou, I.; Mina, P.; Velissariou, V.; Vittas, S.; Santana, A.; Alexidis, F.; Tsangaras, K. Non-invasive prenatal testing of fetal chromosomal aneuploidies: validation and clinical performance of the veracity test. Molecular Cytogenetics 2019, 12, 1-7.

48. Langlois, S.; Johnson, J.; Audibert, F.; Gekas, J.; Forest, J.C.; Caron, A.; Harrington, K.; Pastuck, M.; Meddour, H.; Tétu, A. Comparison of first-tier cell-free DNA screening for common aneuploidies with conventional publically funded screening. Prenatal Diagnosis 2017, 37, 1238-1244.

49. Lau, T.K.; Chen, F.; Pan, X.; Pooh, R.K.; Jiang, F.; Li, Y.; Jiang, H.; Li, X.; Chen, S.; Zhang, X. Non-invasive prenatal diagnosis of common fetal chromosomal aneuploidies by maternal plasma DNA sequencing. The Journal of Maternal-Fetal & Neonatal Medicine 2012, 25, 1370-1374.

50. Lee, D.E.; Kim, H.; Park, J.; Yun, T.; Park, D.Y.; Kim, M.; Ryu, H.M. Clinical Validation of Non-Invasive Prenatal Testing for Fetal Common Aneuploidies in 1,055 Korean Pregnant Women: a Single Center Experience. Journal of Korean Medical Science 2019, 34.

51. Liang, D.; Lv, W.; Wang, H.; Xu, L.; Liu, J.; Li, H.; Hu, L.; Peng, Y.; Wu, L. Non-invasive prenatal testing of fetal whole chromosome aneuploidy by massively parallel sequencing. Prenatal Diagnosis 2013, 33, 409-415.

52. Mazloom, A.R.; Džakula, Ž.; Oeth, P.; Wang, H.; Jensen, T.; Tynan, J.; McCullough, R.; Saldivar, J.S.; Ehrich, M.; van den Boom, D. Noninvasive prenatal detection of sex chromosomal aneuploidies by sequencing circulating cell-free DNA from maternal plasma. Prenatal Diagnosis 2013, 33, 591-597.

53. Miltoft, C.; Rode, L.; Ekelund, C.; Sundberg, K.; Kjaergaard, S.; Zingenberg, H.; Tabor, A. Contingent first-trimester screening for aneuploidies with cell-free DNA in a Danish clinical setting. Ultrasound in Obstetrics & Gynecology 2018, 51, 470-479.

54. Nicolaides, K.H.; Syngelaki, A.; Ashoor, G.; Birdir, C.; Touzet, G. Noninvasive prenatal testing for fetal trisomies in a routinely screened first-trimester population. American Journal of Obstetrics and Gy-necology 2012, 207, 374. e371-374. e376.

55. Nicolaides, K.; Syngelaki, A.; Gil, M.; Atanasova, V.; Markova, D. Validation of targeted sequencing of single-nucleotide polymorphisms for non-invasive prenatal detection of aneuploidy of chromosomes 13, 18, 21, X, and Y. Prenatal Diagnosis 2013, 33, 575-579.

56. Nicolaides, K.H.; Syngelaki, A.; Gil, M.d.M.; Quezada, M.S.; Zinevich, Y. Prenatal detection of fetal triploidy from cell-free DNA testing in maternal blood. Fetal Diagnosis and Therapy 2014, 35, 212-217.

57. Norton, M.E.; Brar, H.; Weiss, J.; Karimi, A.; Laurent, L.C.; Caughey, A.B.; Rodriguez, M.H.; Williams III, J.; Mitchell, M.E.; Adair, C.D. Non-Invasive Chromosomal Evaluation (NICE) Study: results of a multicenter prospective cohort study for detection of fetal trisomy 21 and trisomy 18. American Journal of Obstetrics and Gynecology 2012, 207, 137. e131-137. e138.

58. Norton, M.E.; Jacobsson, B.; Swamy, G.K.; Laurent, L.C.; Ranzini, A.C.; Brar, H.; Tomlinson, M.W.; Pereira, L.; Spitz, J.L.; Hollemon, D. Cell-free DNA analysis for noninvasive examination of trisomy. New England Journal of Medicine 2015, 372, 1589-1597.

59. Palomaki, G.E.; Deciu, C.; Kloza, E.M.; Lambert-Messerlian, G.M.; Haddow, J.E.; Neveux, L.M.; Ehrich, M.; van den Boom, D.; Bombard, A.T.; Grody, W.W. DNA sequencing of maternal plasma reliably identifies trisomy 18 and trisomy 13 as well as Down syndrome: an international collaborative study. Genetics in Medicine 2012, 14, 296-305.

60. Papageorghiou, A.T.; Khalil, A.; Forman, M.; Hulme, R.; Mazey, R.; Mousa, H.A.; Johnstone, E.D.; McKelvey, A.; Cohen, K.E.; Risley, M. Clinical evaluation of the IONA test: a non-invasive prenatal screening test for trisomies 21, 18 and 13. Ultrasound in Obstetrics & Gynecology 2016, 47, 188-193.

61. Persico, N.; Boito, S.; Ischia, B.; Cordisco, A.; De Robertis, V.; Fabietti, I.; Periti, E.; Volpe, P.; Fedele, L.; Rembouskos, G. Cell-free DNA testing in the maternal blood in high-risk pregnancies after first-trimester combined screening. Prenatal Diagnosis 2016, 36, 232-236.

62. Persico, N.; Boito, S.; Volpe, P.; Ischia, B.; Gentile, M.; Ronzoni, L.; De Robertis, V.; Fabietti, I.; Olivieri, C.; Periti, E. Incidence of chromosomal abnormalities in fetuses with first trimester ultrasound anom-alies and a low-risk cell-free DNA test for common trisomies. Prenatal Diagnosis 2020, 40, 1474-1481.

63. Pertile, M.D.; Flowers, N.; Vavrek, D.; Andrews, D.; Kalista, T.; Craig, A.; Deciu, C.; Duenwald, S.; Meier, K.; Bhatt, S. Performance of a paired-end sequencing-based noninvasive prenatal screening test in the detection of genome-wide fetal chromosomal anomalies. Clinical Chemistry 2021, 67, 1210-1219.

64. Pooh, R.K.; Masuda, C.; Matsushika, R.; Machida, M.; Nakamura, T.; Takeda, M.; Ohashi, H.; Kumagai, M.; Uenishi, K.; Roos, F. Clinical Validation of Fetal cfDNA Analysis Using Roll-ing-Circle-Replication and Imaging Technology in Osaka (CRITO Study). Diagnostics 2021, 11, 1837.

65. Poon, L.; Dumidrascu-Diris, D.; Francisco, C.; Fantasia, I.; Nicolaides, K. IONA test for first-trimester detection of trisomies 21, 18 and 13. Ultrasound in Obstetrics & Gynecology 2016, 47, 184-187.

66. Porreco, R.P.; Garite, T.J.; Maurel, K.; Marusiak, B.; Network, O.C.R.; Ehrich, M.; van den Boom, D.; Deciu, C.; Bombard, A. Noninvasive prenatal screening for fetal trisomies 21, 18, 13 and the common sex chromosome aneuploidies from maternal blood using massively parallel genomic sequencing of DNA. American Journal of Obstetrics and Gynecology 2014, 211, 365. e361-365. e312.

67. Porreco, R.P.; Sekedat, M.; Bombard, A.; Garite, T.J.; Maurel, K.; Marusiak, B.; Adair, D.; Bleich, A.; Combs, C.A.; Kramer, W. Evaluation of a novel screening method for fetal aneuploidy using cell-free DNA in maternal plasma. Journal of Medical Screening 2020, 27, 1-8.

68. Ryan, A.; Hunkapiller, N.; Banjevic, M.; Vankayalapati, N.; Fong, N.; Jinnett, K.N.; Demko, Z.; Zim-mermann, B.; Sigurjonsson, S.; Gross, S.J. Validation of an enhanced version of a single-nucleotide

polymorphism-based noninvasive prenatal test for detection of fetal aneuploidies. Fetal diagnosis and Therapy 2016, 40, 219-223.

69. Sehnert, A.J.; Rhees, B.; Comstock, D.; de Feo, E.; Heilek, G.; Burke, J.; Rava, R.P. Optimal detection of fetal chromosomal abnormalities by massively parallel DNA sequencing of cell-free fetal DNA from maternal blood. Clinical Chemistry 2011, 57, 1042-1049.

70. Shaw, S.S.; Hsiao, C.-H.; Chen, C.-Y.; Ren, Y.; Tian, F.; Tsai, C.; Chen, M.; Cheng, P.-J. Noninvasive prenatal testing for whole fetal chromosomal aneuploidies: a multicenter prospective cohort trial in Taiwan. Fetal Diagnosis and Therapy 2014, 35, 13-17.

71. Song, Y.; Liu, C.; Qi, H.; Zhang, Y.; Bian, X.; Liu, J. Noninvasive prenatal testing of fetal aneuploidies by massively parallel sequencing in a prospective Chinese population. Prenatal Diagnosis 2013, 33, 700-706.

72. Song, Y.; Huang, S.; Zhou, X.; Jiang, Y.; Qi, Q.; Bian, X.; Zhang, J.; Yan, Y.; Cram, D.; Liu, J. Non-invasive prenatal testing for fetal aneuploidies in the first trimester of pregnancy. Ultrasound in Obstetrics & Gynecology 2015, 45, 55-60.

73. Stokowski, R.; Wang, E.; White, K.; Batey, A.; Jacobsson, B.; Brar, H.; Balanarasimha, M.; Hollemon, D.; Sparks, A.; Nicolaides, K. Clinical performance of non-invasive prenatal testing (NIPT) using targeted cell-free DNA analysis in maternal plasma with microarrays or next generation sequencing (NGS) is consistent across multiple controlled clinical studies. Prenatal Diagnosis 2015, 35, 1243-1246.

74. Stumm, M.; Entezami, M.; Haug, K.; Blank, C.; Wüstemann, M.; Schulze, B.; Raabe-Meyer, G.; Hempel, M.; Schelling, M.; Ostermayer, E. Diagnostic accuracy of random massively parallel sequencing for non-invasive prenatal detection of common autosomal aneuploidies: a collaborative study in Europe. Prenatal Diagnosis 2014, 34, 185-191.

75. Tsaliki, E.; Papageorgiou, E.A.; Spyrou, C.; Koumbaris, G.; Kypri, E.; Kyriakou, S.; Sotiriou, C.; Touvana, E.; Keravnou, A.; Karagrigoriou, A. MeDIP real-time qPCR of maternal peripheral blood reliably identifies trisomy 21. Prenatal Diagnosis 2012, 32, 996-1001.

76. Tynan, J.; Kim, S.; Mazloom, A.; Zhao, C.; McLennan, G.; Tim, R.; Liu, L.; Hannum, G.; Hull, A.; Bombard, A. Application of risk score analysis to low-coverage whole genome sequencing data for the noninvasive detection of trisomy 21, trisomy 18, and trisomy 13. Prenatal Diagnosis 2016, 36, 56-62.

77. Verweij, E.; Jacobsson, B.; Van Scheltema, P.; de Boer, M.; Hoffer, M.; Hollemon, D.; Westgren, M.; Song, K.; Oepkes, D. European non-invasive trisomy evaluation (EU-NITE) study: a multicenter prospective cohort study for non-invasive fetal trisomy 21 testing. Prenatal Diagnosis 2013, 33, 996-1001.

78. Alyafee, Y.; Al Tuwaijri, A.; Alam, Q.; Umair, M.; Haddad, S.; Alharbi, M.; Ballow, M.; Al Drees, M.; AlAbdulrahman, A.; Al Khaldi, A.; et al. Next Generation Sequencing Based Non-invasive Prenatal

Testing (NIPT): First Report From Saudi Arabia. Front Genet 2021, 12, 630787, doi:10.3389/fgene.2021.630787.

79. Bajka, A.; Bajka, M.; Chablais, F.; Burkhardt, T. Audit of the first > 7500 noninvasive prenatal aneuploidy tests in a Swiss genetics center. Arch Gynecol Obstet 2022, 305, 1185-1192, doi:10.1007/s00404-021-06203-7.

80. Beamon, C.J.; Hardisty, E.E.; Harris, S.C.; Vora, N.L. A single center's experience with noninvasive prenatal testing. Genet Med 2014, 16, 681-687, doi:10.1038/gim.2014.20.

81. Bianchi, D.W.; Parsa, S.; Bhatt, S.; Halks-Miller, M.; Kurtzman, K.; Sehnert, A.J.; Swanson, A. Fetal sex chromosome testing by maternal plasma DNA sequencing: clinical laboratory experience and biology. Obstetrics & Gynecology 2015, 125, 375-382, doi:10.1097/AOG.0000000000000637.

82. Borth, H.; Teubert, A.; Glaubitz, R.; Knippenberg, S.; Kutur, N.; Winkler, T.; Eiben, B. Analysis of cell-free DNA in a consecutive series of 13,607 routine cases for the detection of fetal chromosomal aneuploidies in a single center in Germany. Arch Gynecol Obstet 2021, 303, 1407-1414, doi:10.1007/s00404-020-05856-0.

83. Bu, J.; Jiang, P.; Cui, X.; Zhou, H.; Han, F. Application values of prenatal screening and non-invasive gene sequencing in fetal birth defects. Pak J Med Sci 2020, 36, 1545-1549, doi:10.12669/pjms.36.7.2290.

84. Chen, Y.; Yu, Q.; Mao, X.; Lei, W.; He, M.; Lu, W. Noninvasive prenatal testing for chromosome aneuploidies and subchromosomal microdeletions/microduplications in a cohort of 42,910 single pregnancies with different clinical features. Hum Genomics 2019, 13, 60, doi:10.1186/s40246-019-0250-2.

85. Comas, C.; Echevarria, M.; Rodriguez, M.A.; Prats, P.; Rodriguez, I.; Serra, B. Initial experience with non-invasive prenatal testing of cell-free DNA for major chromosomal anomalies in a clinical setting. J Matern Fetal Neonatal Med 2015, 28, 1196-1201, doi:10.3109/14767058.2014.947579.

86. Dar, P.; Curnow, K.J.; Gross, S.J.; Hall, M.P.; Stosic, M.; Demko, Z.; Zimmermann, B.; Hill, M.; Sigurjonsson, S.; Ryan, A.; et al. Clinical experience and follow-up with large scale single-nucleotide polymorphism-based noninvasive prenatal aneuploidy testing. American Journal of Obstetrics and Gynecology 2014, 211, 527 e521-527 e517, doi:10.1016/j.ajog.2014.08.006.

87. Dai, R.; Yu, Y.; Zhang, H.; Li, L.; Jiang, Y.; Liu, R.; Zhang, H. Analysis of 17,428 pregnant women undergoing non-invasive prenatal testing for fetal chromosome in Northeast China. Medicine (Baltimore) 2021, 100, e24740, doi:10.1097/MD.0000000000024740.

88. Dan, S.; Wang, W.; Ren, J.; Li, Y.; Hu, H.; Xu, Z.; Lau, T.K.; Xie, J.; Zhao, W.; Huang, H.; et al. Clinical application of massively parallel sequencing-based prenatal noninvasive fetal trisomy test for trisomies 21 and 18 in 11,105 pregnancies with mixed risk factors. Prenatal Diagnosis 2012, 32, 1225-1232, doi:10.1002/pd.4002.

89. Du, Y.; Lin, J.; Lan, L.; Dong, Y.; Zhu, J.; Jiang, W.; Pan, X.; Lu, Y.; Li, D.; Wang, L. Detection of chromosome abnormalities using current noninvasive prenatal testing: A multi-center comparative study. Biosci Trends 2018, 12, 317-324, doi:10.5582/bst.2018.01044.90.

90. Eiben, B.; Krapp, M.; Borth, H.; Kutur, N.; Kreiselmaier, P.; Glaubitz, R.; Deutinger, J.; Merz, E. Single Nucleotide Polymorphism-Based Analysis of Cell-Free Fetal DNA in 3000 Cases from Germany and Austria. *Ultrasound Int Open* **2015**, *1*, E8-E11, doi:10.1055/s-0035-1555765.

91. Fairbrother, G.; Johnson, S.; Musci, T.J.; Song, K. Clinical experience of noninvasive prenatal testing with cell-free DNA for fetal trisomies 21, 18, and 13, in a general screening population. *Prenat Diagn* **2013**, *33*, 580-583, doi:10.1002/pd.4092.

92. Fiorentino, F.; Bono, S.; Pizzuti, F.; Duca, S.; Polverari, A.; Faieta, M.; Baldi, M.; Diano, L.; Spinella, F. The clinical utility of genome-wide non invasive prenatal screening. *Prenatal Diagnosis* **2017**, *37*, 593-601, doi:10.1002/pd.5053.

93. Futch, T.; Spinosa, J.; Bhatt, S.; de Feo, E.; Rava, R.P.; Sehnert, A.J. Initial clinical laboratory experience in noninvasive prenatal testing for fetal aneuploidy from maternal plasma DNA samples. *Prenatal Diagnosis* **2013**, *33*, 569-574, doi:10.1002/pd.4123.

94. Garshasbi, M.; Wang, Y.; Hantoosh Zadeh, S.; Giti, S.; Piri, S.; Reza Hekmat, M. Clinical Application of Cell-Free DNA Sequencing-Based Noninvasive Prenatal Testing for Trisomies 21, 18, 13 and Sex Chromosome Aneuploidy in a Mixed-Risk Population in Iran. *Fetal Diagn Ther* **2020**, *47*, 220-227, doi:10.1159/000501014.

95. Ge, Y.; Li, J.; Zhuang, J.; Zhang, J.; Huang, Y.; Tan, M.; Li, W.; Chen, J.; Zhou, Y. Expanded noninvasive prenatal testing for fetal aneuploidy and copy number variations and parental willingness for invasive diagnosis in a cohort of 18,516 cases. *BMC Med Genomics* **2021**, *14*, 106, doi:10.1186/s12920-021-00955-6.

96. Gil, M.M.; Quezada, M.S.; Bregant, B.; Ferraro, M.; Nicolaides, K.H. Implementation of maternal blood cell-free DNA testing in early screening for aneuploidies. *Ultrasound Obstetrics & Gynecology* **2013**, *42*, 34-40, doi:10.1002/uog.12504.

97. Gil, M.M.; Brik, M.; Casanova, C.; Martin-Alonso, R.; Verdejo, M.; Ramirez, E.; Santacruz, B. Screening for trisomies 21 and 18 in a Spanish public hospital: from the combined test to the cell-free DNA test. *J Matern Fetal Neonatal Med* **2017**, *30*, 2476-2482, doi:10.1080/14767058.2016.1253062.

98. Guy, C.; Haji-Sheikhi, F.; Rowland, C.M.; Anderson, B.; Owen, R.; Lacbawan, F.L.; Alagia, D.P. Prenatal cell-free DNA screening for fetal aneuploidy in pregnant women at average or high risk: Results from a large US clinical laboratory. *Mol Genet Genomic Med* **2019**, *7*, e545, doi:10.1002/mgg3.545.

99. Guy, G.P.; Hargrave, J.; Dunn, R.; Price, K.; Short, J.; Thilaganathan, B.; collaborative, S.t. Secondary non-invasive prenatal screening for fetal trisomy: an effectiveness study in a public health setting. *BJOG* **2021**, *128*, 440-446, doi:10.1111/1471-0528.16464.

100. Harasim, T.; Neuhann, T.; Behnecke, A.; Stampfer, M.; Holinski-Feder, E.; Abicht, A. Initial Clinical Experience with NIPT for Rare Autosomal Aneuploidies and Large Copy Number Variations. *J Clin Med* **2022**, *11*, doi:10.3390/jcm11020372.

101. Hong, S.Y.; Shim, S.H.; Park, H.J.; Shim, S.S.; Kim, J.Y.; Cho, Y.K.; Kim, S.H.; Cha, D.H. Experiences and efficacy of noninvasive prenatal test using maternal plasma in single center: 1,591 cases. *J Genet Med* **2020**, *17*, 11-15, doi:10.5734/JGM.2020.17.1.11.

102. Hu, H.; Wang, L.; Wu, J.; Zhou, P.; Fu, J.; Sun, J.; Cai, W.; Liu, H.; Yang, Y. Noninvasive prenatal testing for chromosome aneuploidies and subchromosomal microdeletions/microduplications in a cohort of 8141 single pregnancies. *Hum Genomics* **2019**, *13*, 14, doi:10.1186/s40246-019-0198-2.

103. Jackson, J.; Hamar, B.; Lazar, E.; Lim, K.; Rodriguez, D.; Stock, K.; Wolfberg, A.J.; Dunk, R. Nuchal translucency measurement plus non-invasive prenatal testing to screen for aneuploidy in a community-based average-risk population. *Ultrasound Obstetrics & Gynecology* **2014**, *44*, 491, doi:10.1002/uog.13424.

104. Kagan, K.O.; Hoopmann, M.; Pfaff, T.; Prodan, N.; Wagner, P.; Schmid, M.; Dufke, A.; Mau-Holzmann, U.; Brucker, S.; Marcato, L.; et al. First Trimester Screening for Common Trisomies and Microdeletion 22q11.2 Syndrome Using Cell-Free DNA: A Prospective Clinical Study. *Fetal Diagn Ther* **2020**, *47*, 841-852, doi:10.1159/000510069.

105. Koc, A.; Ozer Kaya, O.; Ozyilmaz, B.; Kutbay, Y.B.; Kirbiyik, O.; Ozdemir, T.R.; Erdogan, K.M.; Saka Guvenc, M.; Oztekin, D.C.; Ozeren, M.; et al. Targeted fetal cell-free DNA screening for aneuploidies in 4,594 pregnancies: Single center study. *Mol Genet Genomic Med* **2019**, *7*, e00678, doi:10.1002/mgg3.678.

106. Korostelev, S.; Totchiev, G.; Kanivets, I.; Gnetetskaya, V. Association of non-invasive prenatal testing and chromosomal microarray analysis for prenatal diagnostics. *Gynecol Endocrinol* **2014**, *30 Suppl 1*, 13-16, doi:10.3109/09513590.2014.945770.

107. La Verde, M.; De Falco, L.; Torella, A.; Savarese, G.; Savarese, P.; Ruggiero, R.; Conte, A.; Fico, V.; Torella, M.; Fico, A. Performance of cell-free DNA sequencing-based non-invasive prenatal testing: experience on 36,456 singleton and multiple pregnancies. *BMC Med Genomics* **2021**, *14*, 93, doi:10.1186/s12920-021-00941-y.

108. Lai, Y.; Zhu, X.; He, S.; Dong, Z.; Tang, Y.; Xu, F.; Chen, Y.; Meng, L.; Tao, Y.; Yi, S.; et al. Performance of Cell-Free DNA Screening for Fetal Common Aneuploidies and Sex Chromosomal Abnormalities: A Prospective Study from a Less Developed Autonomous Region in Mainland China. *Genes (Basel)* **2021**, *12*, doi:10.3390/genes12040478.

109. Lau, T.K.; Cheung, S.W.; Lo, P.S.; Pursley, A.N.; Chan, M.K.; Jiang, F.; Zhang, H.; Wang, W.; Jong, L.F.; Yuen, O.K.; et al. Non-invasive prenatal testing for fetal chromosomal abnormalities by low-coverage whole-genome sequencing of maternal plasma DNA: review of 1982 consecutive cases in a single center. *Ultrasound Obstet Gynecol* **2014**, *43*, 254-264, doi:10.1002/uog.13277.

110. Liang, D.; Cram, D.S.; Tan, H.; Linpeng, S.; Liu, Y.; Sun, H.; Zhang, Y.; Tian, F.; Zhu, H.; Xu, M.; et al. Clinical utility of noninvasive prenatal screening for expanded chromosome disease syndromes. *Genet Med* **2019**, *21*, 1998-2006, doi:10.1038/s41436-019-0467-4.

111. Liu, Y.; Liu, H.; He, Y.; Xu, W.; Ma, Q.; He, Y.; Lei, W.; Chen, G.; He, Z.; Huang, J.; et al. Clinical performance of non-invasive prenatal served as a first-tier screening test for trisomy 21, 18, 13 and sex chromosome aneuploidy in a pilot city in China. *Hum Genomics* **2020**, *14*, 21, doi:10.1186/s40246-020-00268-2.

112. Lu, W.; Huang, T.; Wang, X.R.; Zhou, J.H.; Yuan, H.Z.; Yang, Y.; Huang, T.T.; Liu, D.P.; Liu, Y.Q. Next-generation sequencing: a follow-up of 36,913 singleton pregnancies with noninvasive prenatal testing in central China. *J Assist Reprod Genet* **2020**, *37*, 3143-3150, doi:10.1007/s10815-020-01977-2.

113. Lu, X.; Wang, C.; Sun, Y.; Tang, J.; Tong, K.; Zhu, J. Noninvasive prenatal testing for assessing foetal sex chromosome aneuploidy: a retrospective study of 45,773 cases. *Mol Cytogenet* **2021**, *14*, 1, doi:10.1186/s13039-020-00521-2.

114. Lu, Y.; Zhou, S.; Linpeng, S.; Ding, S.; Li, S.; Li, Y.; Shi, L.; He, J.; Liu, Y. Cell-Free DNA Screening for Sex Chromosome Abnormalities and Pregnancy Outcomes, 2018-2020: A Retrospective Analysis. *J Pers Med* **2022**, *12*, doi:10.3390/jpm12010048.

115. Lund, I.C.B.; Petersen, O.B.; Becher, N.H.; Lildballe, D.L.; Jorgensen, F.S.; Ambye, L.; Skibsted, L.; Ernst, A.; Jensen, A.N.; Fagerberg, C.; et al. National data on the early clinical use of non-invasive prenatal testing in public and private healthcare in Denmark 2013-2017. *Acta Obstet Gynecol Scand* **2021**, *100*, 884-892, doi:10.1111/aogs.14052.

116. Luo, Y.; Hu, H.; Jiang, L.; Ma, Y.; Zhang, R.; Xu, J.; Pan, Y.; Long, Y.; Yao, H.; Liang, Z. A retrospective analysis the clinic data and follow-up of non-invasive prenatal test in detection of fetal chromosomal aneuploidy in more than 40,000 cases in a single prenatal diagnosis center. *Eur J Med Genet* **2020**, *63*, 104001, doi:10.1016/j.ejmg.2020.104001.

117. Luo, Y.; Hu, H.; Zhang, R.; Ma, Y.; Pan, Y.; Long, Y.; Hu, B.; Yao, H.; Liang, Z. An assessment of the analytical performance of non-invasive prenatal testing (NIPT) in detecting sex chromosome aneuploidies: 34,717-patient sample in a single prenatal diagnosis Centre in China. *J Gene Med* **2021**, *23*, e3362, doi:10.1002/jgm.3362.

118. Luthgens, K.; Grati, F.R.; Sinzel, M.; Habig, K.; Kagan, K.O. Confirmation rate of cell free DNA screening for sex chromosomal abnormalities according to the method of confirmatory testing. *Prenatal Diagnosis* **2021**, *41*, 1258-1263, doi:10.1002/pd.5814.

119. Manotaya, S.; Xu, H.; Uerpairojkit, B.; Chen, F.; Charoenvidhya, D.; Liu, H.; Petcharaburanin, N.; Liu, Y.; Tang, S.; Wang, X.; et al. Clinical experience from Thailand: noninvasive prenatal testing as screening tests for trisomies 21, 18 and 13 in 4736 pregnancies. *Prenatal Diagnosis* **2016**, *36*, 224-231, doi:10.1002/pd.4775.

120. Margiotti, K.; Cesta, A.; Dello Russo, C.; Cima, A.; Barone, M.A.; Viola, A.; Sparacino, D.; Mesoraca, A.; Giorlandino, C. Cell-free DNA screening for sex chromosomal aneuploidies in 9985 pregnancies: Italian single experience. *BMC Res Notes* **2020**, *13*, 167, doi:10.1186/s13104-020-05009-1.

121. Mesoraca, A.; Margiotti, K.; Dello Russo, C.; Cesta, A.; Cima, A.; Longo, S.A.; Barone, M.A.; Viola, A.; Sparacino, D.; Giorlandino, C. Cell-free DNA screening for aneuploidies in 7113 pregnancies: single Italian centre study. *Genet Res (Camb)* **2020**, *102*, e5, doi:10.1017/S001667232000004X.

122. Noh, J.J.; Ryu, H.M.; Oh, S.Y.; Choi, S.J.; Roh, C.R.; Kim, J.H. A two-year experience of non-invasive prenatal testing (NIPT) at an urban tertiary medical center in South Korea. *Taiwan J Obstet Gynecol* **2019**, *58*, 545-551, doi:10.1016/j.tjog.2019.05.021.

123. Oepkes, D.; Page-Christiaens, G.C.; Bax, C.J.; Bekker, M.N.; Bilardo, C.M.; Boon, E.M.; Schuring-Blom, G.H.; Coumans, A.B.; Faas, B.H.; Galjaard, R.H.; et al. Trial by Dutch laboratories for evaluation of non-invasive prenatal testing. Part I-clinical impact. *Prenatal Diagnosis* **2016**, *36*, 1083-1090, doi:10.1002/pd.4945.

124. Oneda, B.; Sirleto, P.; Baldinger, R.; Taralczak, M.; Joset, P.; Zweier, M.; Niedrist, D.; Azzarello-Burri, S.; Britschgi, C.; Breymann, C.; et al. Genome-wide non-invasive prenatal testing in single- and multiple-pregnancies at any risk: Identification of maternal polymorphisms to reduce the number of unnecessary invasive confirmation testing. *Eur J Obstet Gynecol Reprod Biol* **2020**, *252*, 19-29, doi:10.1016/j.ejogrb.2020.05.070.

125. Palomaki, G.E.; Kloza, E.M.; O'Brien, B.M.; Eklund, E.E.; Lambert-Messerlian, G.M. The clinical utility of DNA-based screening for fetal aneuploidy by primary obstetrical care providers in the general pregnancy population. *Genetics in Medicine* **2017**, *19*, 778-786, doi:10.1038/gim.2016.194.

126. Panchalee, T.; Poungvarin, N.; Amornrit, W.; Pooliam, J.; Taluengjit, P.; Wataganara, T. Clinical performance of DNA-based prenatal screening using single-nucleotide polymorphisms approach in Thai women with singleton pregnancy. *Mol Genet Genomic Med* **2020**, *8*, e1256, doi:10.1002/mgg3.1256.

127. Pang, Y.; Wang, C.; Tang, J.; Zhu, J. Clinical application of noninvasive prenatal testing in the detection of fetal chromosomal diseases. *Molecular Cytogenetics* **2021**, *14*, 31, doi:10.1186/s13039-021-00550-5.

128. Pavanello, E.; Sciarrone, A.; Guaraldo, V.; Muccinelli, E.; Ciuffreda, V.P.; Sauro, P.; Bondielli, G.; Mirante, S.; Mengozzi, G.; Viora, E.; et al. Cell-free DNA screening for fetal aneuploidy using the rolling circle method: A step towards non invasive prenatal testing simplification. *Prenatal Diagnosis* **2021**, *41*, 1694-1700, doi:10.1002/pd.6050.

129. Pescia, G.; Guex, N.; Iseli, C.; Brennan, L.; Osteras, M.; Xenarios, I.; Farinelli, L.; Conrad, B. Cell-free DNA testing of an extended range of chromosomal anomalies: clinical experience with 6,388 consecutive cases. *Genetics in Medicine* **2017**, *19*, 169-175, doi:10.1038/gim.2016.72.

130. Qi, Q.G.; Tuo, Y.; Liu, L.X.; Yu, C.X.; Wu, A.N. Amniocentesis and Next Generation Sequencing (NGS)-Based Noninvasive Prenatal DNA Testing (NIPT) for Prenatal Diagnosis of Fetal Chromosomal Disorders. *Int J Gen Med* **2021**, *14*, 1811-1817, doi:10.2147/IJGM.S297585.

131. Quezada, M.S.; Gil, M.M.; Francisco, C.; Orosz, G.; Nicolaides, K.H. Screening for trisomies 21, 18 and 13 by cell-free DNA analysis of maternal blood at 10-11 weeks' gestation and the combined test at 11-13 weeks. *Ultrasound Obstetrics & Gynecology* **2015**, *45*, 36-41, doi:10.1002/uog.14664.

132. Radoi, V.E.; Bohiltea, C.L.; Bohiltea, R.E.; Albu, D.N. Cell free fetal DNA testing in maternal blood of Romanian pregnant women. *Iran J Reprod Med* **2015**, *13*, 623-626.

133. Sainz, J.A.; Torres, M.R.; Peral, I.; Granell, R.; Vargas, M.; Carrasco, P.; Garcia-Mejido, J.A.; Santacruz, B.; Gil, M.M. Clinical and Economic Evaluation after Adopting Contingent Cell-Free DNA Screening for Fetal Trisomies in South Spain. *Fetal Diagn Ther* **2020**, 1-8, doi:10.1159/000508306.

134. Santamaria, R.; Bermejo, B.; Cigarrán, S.; Benn, P. A National Referral Laboratory's Experience with the Implementation of SNP-Based Non-invasive Prenatal Screening for Fetal Aneuploidy and Select Microdeletion Syndromes. *Journal of Fetal Medicine* **2018**, *5*, 7-12, doi:10.1007/s40556-017-0143-1.

135. Sasaki, Y.; Yamada, T.; Tanaka, S.; Sekizawa, A.; Hirose, T.; Suzumori, N.; Kaji, T.; Kawaguchi, S.; Hasuo, Y.; Nishizawa, H.; et al. Evaluation of the clinical performance of noninvasive prenatal testing at a Japanese laboratory. *J Obstet Gynaecol Res* **2021**, *47*, 3437-3446, doi:10.1111/jog.14954.

136. Serapinas, D.; Boreikaite, E.; Bartkeviciute, A.; Norvilaite, K.; Narbekovas, A.; Bartkeviciene, D. The Level of Free Fetal DNA as Precise Noninvasive Marker for Chromosomal Aneuploidies: First Results from BALTIC Region. *Medicina (Kaunas)* **2020**, *56*, doi:10.3390/medicina56110579.

137. Song, J.P.; Jiang, Y.F.; Gao, T.X.; Yao, Y.Y.; Liu, L.J.; Xu, R.H.; Yi, M.Q.; Yu, C.J.; Wang, W.P.; Li, H. Performance of non-invasive prenatal screening for sex chromosome aneuploidies and parental decision-making. *Chin Med J (Engl)* **2020**, *133*, 1617-1619, doi:10.1097/CM9.0000000000000868.

138. Strom, C.M.; Anderson, B.; Tsao, D.; Zhang, K.; Liu, Y.; Livingston, K.; Elzinga, C.; Evans, M.; Nguyen, Q.; Wolfson, D. Improving the positive predictive value of non-invasive prenatal screening (NIPS). *PloS One* **2017**, *12*, e0167130.

139. Togneri, F.S.; Kilby, M.D.; Young, E.; Court, S.; Williams, D.; Griffiths, M.J.; Allen, S.K. Implementation of cell-free DNA-based non-invasive prenatal testing in a National Health Service Regional Genetics Laboratory. *Genet Res (Camb)* **2019**, *101*, e11, doi:10.1017/S0016672319000119.

140. van der Meij, K.R.M.; Sistermans, E.A.; Macville, M.V.E.; Stevens, S.J.C.; Bax, C.J.; Bekker, M.N.; Bilardo, C.M.; Boon, E.M.J.; Boter, M.; Diderich, K.E.M.; et al. TRIDENT-2: National Implementation of Genome-wide Non-invasive Prenatal Testing as a First-Tier Screening Test in the Netherlands. *Am J Hum Genet* **2019**, *105*, 1091-1101, doi:10.1016/j.ajhg.2019.10.005.

141. Verma, I.C.; Puri, R.; Venkataswamy, E.; Tayal, T.; Nampoorthiri, S.; Andrew, C.; Kabra, M.; Bagga, R.; Gowda, M.; Batra, M.; et al. Single Nucleotide Polymorphism-Based Noninvasive Prenatal Testing: Experience in India. *J Obstet Gynaecol India* **2018**, *68*, 462-470, doi:10.1007/s13224-017-1061-9.

142. Wan, J.H.; Zhen, L.; Han, J.; Pan, M.; Yang, X.; Li, D.Z. Use of noninvasive prenatal screening with cell-free DNA in late pregnancy with sonographic soft markers. *Eur J Obstet Gynecol Reprod Biol* **2020**, *252*, 431-433, doi:10.1016/j.ejogrb.2020.07.036.

143. Wang, Y.; Li, S.; Wang, W.; Dong, Y.; Zhang, M.; Wang, X.; Yin, C. Cell-free DNA screening for sex chromosome aneuploidies by non-invasive prenatal testing in maternal plasma. *Mol Cytogenet* **2020**, *13*, 10, doi:10.1186/s13039-020-0478-5.

144. Wang, C.; Tang, J.; Tong, K.; Huang, D.; Tu, H.; Li, Q.; Zhu, J. Expanding the application of non-invasive prenatal testing in the detection of foetal chromosomal copy number variations. *BMC Med Genomics* **2021**, *14*, 292, doi:10.1186/s12920-021-01131-6.

145. Wang, J.W.; Lyu, Y.N.; Qiao, B.; Li, Y.; Zhang, Y.; Dhanyamraju, P.K.; Bamme, Y.; Yu, M.D.; Yang, D.; Tong, Y.Q. Cell-free fetal DNA testing and its correlation with prenatal indications. *BMC Pregnancy Childbirth* **2021**, *21*, 585, doi:10.1186/s12884-021-04044-5.

146. Willems, P.J.; Dierickx, H.; Vandenakker, E.; Bekedam, D.; Segers, N.; Deboulle, K.; Vereecken, A. The first 3,000 Non-Invasive Prenatal Tests (NIPT) with the Harmony test in Belgium and the Netherlands. *Facts Views Vis Obgyn* **2014**, *6*, 7-12.

147. Xu, L.; Huang, H.; Lin, N.; Wang, Y.; He, D.; Zhang, M.; Chen, M.; Chen, L.; Lin, Y. Non-invasive cell-free fetal DNA testing for aneuploidy: multicenter study of 31 515 singleton pregnancies in southeastern China. *Ultrasound Obstetrics & Gynecology* **2020**, *55*, 242-247, doi:10.1002/uog.20416.

148. Xu, Y.; Jin, P.; Lei, Y.; Qian, Y.; Xu, Y.; Wang, M.; Jin, J.; Yin, Y.; Dong, M. Clinical Efficiency of Non-invasive Prenatal Screening for Common Trisomies in Low-Risk and Twin Pregnancies. *Front Genet* **2021**, *12*, 661884, doi:10.3389/fgene.2021.661884.

149. Xue, Y.; Zhao, G.; Li, H.; Zhang, Q.; Lu, J.; Yu, B.; Wang, T. Non-invasive prenatal testing to detect chromosome aneuploidies in 57,204 pregnancies. *Mol Cytogenet* **2019**, *12*, 29, doi:10.1186/s13039-019-0441-5.

150. Yang, J.; Hou, Y.; Guo, F.; Peng, H.; Wang, D.; Li, Y.; Oy, H.; Wang, Y.; Lu, J.; Yin, A. Noninvasive prenatal detection of fetal sex chromosome abnormalities using the semiconductor sequencing platform (SSP) in Southern China. *J Assist Reprod Genet* **2021**, *38*, 727-734, doi:10.1007/s10815-020-02056-2.

151. Zhang, H.; Gao, Y.; Jiang, F.; Fu, M.; Yuan, Y.; Guo, Y.; Zhu, Z.; Lin, M.; Liu, Q.; Tian, Z.; et al. Non-invasive prenatal testing for trisomies 21, 18 and 13: clinical experience from 146,958 pregnancies. *Ultrasound Obstet Gynecol* **2015**, *45*, 530-538, doi:10.1002/uog.14792.

152. Zheng, J.; Lu, H.; Li, M.; Guan, Y.; Yang, F.; Xu, M.; Dong, J.; Zhang, Q.; An, N.; Zhou, Y. The Clinical Utility of Non-invasive Prenatal Testing for Pregnant Women With Different Diagnostic Indications. *Front Genet* **2020**, *11*, 624, doi:10.3389/fgene.2020.00624.

153. Zheng, Y.; Wan, S.; Dang, Y.; Song, T.; Chen, B.; Zhang, J. Clinical experience regarding the accuracy of NIPT in the detection of sex chromosome abnormality. *J Gene Med* **2020**, *22*, e3199, doi:10.1002/jgm.3199.

154. Zhu, H.; Jin, X.; Xu, Y.; Zhang, W.; Liu, X.; Jin, J.; Qian, Y.; Dong, M. Efficiency of non-invasive prenatal screening in pregnant women at advanced maternal age. *BMC Pregnancy Childbirth* **2021**, *21*, 86, doi:10.1186/s12884-021-03570-6.

155. Dougan, S.D.; Okun, N.; Bellai-Dussault, K.; Meng, L.; Howley, H.E.; Huang, T.; Reszel, J.; Lanes, A.; Walker, M.C.; Armour, C.M. Performance of a universal prenatal screening program incorporating cell-free fetal DNA analysis in Ontario, Canada. *CMAJ* **2021**, *193*, E1156-E1163, doi:10.1503/cmaj.202456.

156. Gug, C.; Mozos, I.; Ratiu, A.; Tudor, A.; Gorduza, E.V.; Caba, L.; Gug, M.; Cojocariu, C.; Furau, C.; Furau, G.; et al. Genetic Counseling and Management: The First Study to Report NIPT Findings in a Romanian Population. *Medicina (Kaunas)* **2022**, *58*, doi:10.3390/medicina58010079.

157. McCullough, R.M.; Almasri, E.A.; Guan, X.; Geis, J.A.; Hicks, S.C.; Mazloom, A.R.; Deciu, C.; Oeth, P.; Bombard, A.T.; Paxton, B.; et al. Non-invasive prenatal chromosomal aneuploidy testing--clinical experience: 100,000 clinical samples. *PLoS One* **2014**, *9*, e109173, doi:10.1371/journal.pone.0109173.

158. Meck, J.M.; Kramer Dugan, E.; Matyakhina, L.; Aviram, A.; Trunca, C.; Pineda-Alvarez, D.; Aradhya, S.; Klein, R.T.; Cherry, A.M. Noninvasive prenatal screening for aneuploidy: positive predictive values based on cytogenetic findings. *American Journal of Obstetrics and Gynecology* **2015**, *213*, 214 e211-215, doi:10.1016/j.ajog.2015.04.001.

159. Saito, M.; Tokunaka, M.; Goto, M.; Takita, H.; Arakaki, T.; Miyagami, K.; Hamada, S.; Oba, T.; Matsuoka, R.; Sekizawa, A. The role of first-trimester ultrasound screening for women with positive noninvasive prenatal testing results. *J Obstet Gynaecol Res* **2022**, *48*, 328-332, doi:10.1111/jog.15115.

160. Suzumori, N.; Sekizawa, A.; Takeda, E.; Samura, O.; Sasaki, A.; Akaishi, R.; Wada, S.; Hamanoue, H.; Hirahara, F.; Sawai, H.; et al. Retrospective details of false-positive and false-negative results in non-invasive prenatal testing for fetal trisomies 21, 18 and 13. *Eur J Obstet Gynecol Reprod Biol* **2021**, *256*, 75-81, doi:10.1016/j.ejogrb.2020.10.050.

161. Tekesin, I. Cell-free DNA Testing in Routine Practice: Characterisation of a Cohort with Positive Results for Trisomies, Sex Chromosome Anomalies and Microdeletions. *Geburtshilfe Frauenheilkd* **2021**, *81*, 81-89, doi:10.1055/a-1226-6538.

162. Togneri, F.S.; Allen, S.K.; Mann, K.; Holgado, E.; Morgan, S. Cytogenomic results following high-chance non-invasive prenatal testing: a UK national audit. *Genet Res (Camb)* **2020**, *102*, e7, doi:10.1017/S0016672320000087.

163. Valderramos, S.G.; Rao, R.R.; Scibetta, E.W.; Silverman, N.S.; Han, C.S.; Platt, L.D. Cell-free DNA screening in clinical practice: abnormal autosomal aneuploidy and microdeletion results. *American Journal of Obstetrics and Gynecology* **2016**, *215*, 626 e621-626 e610, doi:10.1016/j.ajog.2016.06.039.

164. Van Den Bogaert, K.; Lannoo, L.; Brison, N.; Gatinois, V.; Baetens, M.; Blaumeiser, B.; Boemer, F.; Bourlard, L.; Bours, V.; De Leener, A.; et al. Outcome of publicly funded nationwide first-tier noninvasive prenatal screening. *Genet Med* **2021**, *23*, 1137-1142, doi:10.1038/s41436-021-01101-4.

165. Wang, J.C.; Sahoo, T.; Schonberg, S.; Kopita, K.A.; Ross, L.; Patek, K.; Strom, C.M. Discordant noninvasive prenatal testing and cytogenetic results: a study of 109 consecutive cases. *Genetics in Medicine* **2015**, *17*, 234-236, doi:10.1038/gim.2014.92.

166. Wu, X.; Li, Y.; Xie, X.; Su, L.; Cai, M.; Lin, N.; Du, S.; Xu, L.; Huang, H. Clinical Review of Noninvasive Prenatal Testing: Experience from 551 Pregnancies with Noninvasive Prenatal Testing-Positive Results in a Tertiary Referral Center. *J Mol Diagn* **2020**, *22*, 1469-1475, doi:10.1016/j.jmoldx.2020.09.008.

Supplemental Table S2. Validation study characteristics stratified by NIPT method.

	**DANSR**	**SNP**	**MPSS**
**N**	**13**	**6**	**29**
**Publication Year**			
2011-2014	6 (46.2%)	3 (50%)	17 (58.6%)
2015-2018	5 (38.5%)	2 (33.3%)	8 (27.6%)
2019-2022	2 (15.4%)	1 (16.7%)	4 (13.8%)
**Start Year**			
2003-2009	2 (15.4%)	0 (0%)	11 (37.9%)
2010-2013	2 (15.4%)	1 (16.7%)	9 (31%)
2014-2019	2 (15.4%)	1 (16.7%)	4 (13.8%)
Unspecified	7 (53.8%)	4 (66.7%)	5 (17.2%)
**End Year**			
2009-2012	3 (23.1%)	1 (16.7%)	14 (48.3%)
2013-2016	2 (15.4%)	1 (16.7%)	8 (27.6%)
2017-2020	1 (7.7%)	0 (0%)	2 (6.9%)
Unspecified	7 (53.8%)	4 (66.7%)	5 (17.2%)
**Lab Country**			
United States	11 (84.6%)	6 (100%)	9 (31%)
China	0 (0%)	0 (0%)	7 (24.1%)
South Korea	0 (0%)	0 (0%)	2 (6.9%)
Cyprus	2 (15.4%)	0 (0%)	0 (0%)
France	0 (0%)	0 (0%)	2 (6.9%)
Germany	0 (0%)	0 (0%)	2 (6.9%)
Hong Kong	0 (0%)	0 (0%)	2 (6.9%)
UK	0 (0%)	0 (0%)	2 (6.9%)
Sweden	0 (0%)	0 (0%)	0 (0%)
Switzerland	0 (0%)	0 (0%)	1 (3.4%)
Thailand	0 (0%)	0 (0%)	1 (3.4%)
Australia	0 (0%)	0 (0%)	1 (3.4%)
Japan	0 (0%)	0 (0%)	0 (0%)

	DANSR	SNP	MPSS
Malaysia, Canada	0 (0%)	0 (0%)	0 (0%)
**Population Country**			
UK	3 (23.1%)	1 (16.7%)	2 (6.9%)
International	1 (7.7%)	2 (33.3%)	3 (10.3%)
China	0 (0%)	0 (0%)	5 (17.2%)
United States	1 (7.7%)	0 (0%)	4 (13.8%)
France	0 (0%)	0 (0%)	2 (6.9%)
South Korea	0 (0%)	0 (0%)	2 (6.9%)
Japan	0 (0%)	0 (0%)	1 (3.4%)
HK, Netherlands, UK	0 (0%)	0 (0%)	2 (6.9%)
US, Netherlands, Sweden	1 (7.7%)	0 (0%)	0 (0%)
UK, US	1 (7.7%)	0 (0%)	0 (0%)
Thailand	0 (0%)	0 (0%)	1 (3.4%)
Taiwan	0 (0%)	0 (0%)	1 (3.4%)
Sweden, UK, US	1 (7.7%)	0 (0%)	0 (0%)
Netherlands, Sweden	1 (7.7%)	0 (0%)	0 (0%)
Italy	0 (0%)	1 (16.7%)	0 (0%)
Germany/Switzerland	0 (0%)	0 (0%)	1 (3.4%)
Germany	0 (0%)	0 (0%)	1 (3.4%)
Denmark	1 (7.7%)	0 (0%)	0 (0%)
Canada	1 (7.7%)	0 (0%)	0 (0%)
Australia	0 (0%)	0 (0%)	1 (3.4%)
Unspecified	2 (15.4%)	2 (33.3%)	3 (10.3%)
**Population Country**			
United States Only	1 (7.7%)	0 (0%)	4 (13.8%)
Europe Only	2 (15.4%)	1 (16.7%)	3 (10.3%)
International	6 (46.2%)	4 (66.7%)	9 (31%)
Other	4 (30.8%)	1 (16.7%)	13 (44.8%)
**Gestational Age**			
Median (25%ile, 75%ile)	13.1 (12.7, 15.5)	14.1 (12.8, 15.8)	15.2 (14, 16)

	DANSR	SNP	MPSS
96% at 15-20wks	0 (0%)	0 (0%)	1 (3.4%)
Approx 12 - 14.9	1 (7.7%)	0 (0%)	0 (0%)
Range 11 - 13.9	1 (7.7%)	0 (0%)	0 (0%)
Unspecified	1 (7.7%)	0 (0%)	3 (10.3%)
**Maternal Age**			
Median (25%ile, 75%ile)	34.3 (33, 35.2)	34.3 (32.5, 35.7)	35.4 (35, 36.6)
88% <35	0 (0%)	0 (0%)	1 (3.4%)
Unspecified	1 (7.7%)	1 (16.7%)	3 (10.3%)
**Major Study Groups**			
High risk: FTS, STS, u/s, AMA	3 (23.1%)	0 (0%)	17 (58.6%)
High +Low risk groups combined	1 (7.7%)	3 (50%)	3 (10.3%)
Known aneuploid & euploid mix	1 (7.7%)	0 (0%)	4 (13.8%)
All risk	3 (23.1%)	1 (16.7%)	1 (3.4%)
Unspecified	2 (15.4%)	0 (0%)	0 (0%)
<35	0 (0%)	0 (0%)	1 (3.4%)
>=35	0 (0%)	0 (0%)	1 (3.4%)
Combined test risk >=1000	1 (7.7%)	0 (0%)	0 (0%)
High risk + mixed risk	1 (7.7%)	0 (0%)	0 (0%)
High risk: FTS	0 (0%)	1 (16.7%)	0 (0%)
High risk: FTS, u/s, AMA	0 (0%)	1 (16.7%)	0 (0%)
Mainly high risk	0 (0%)	0 (0%)	1 (3.4%)
Prior NIPT with confirmation	0 (0%)	0 (0%)	1 (3.4%)
**Study Type**			
Retrospective	6 (46.2%)	3 (50%)	11 (37.9%)
Prospective	5 (38.5%)	3 (50%)	13 (44.8%)
Other	2 (15.4%)	0 (0%)	5 (17.2%)

Supplemental Table S3. Validation study characteristics stratified by study design

	Retrospective	Prospective	Other
**N**	**22**	**24**	**9**
**Publication Year**			
2011-2014	13 (59.1%)	10 (41.7%)	5 (55.6%)
2015-2018	6 (27.3%)	8 (33.3%)	2 (22.2%)
2019-2022	3 (13.6%)	6 (25%)	2 (22.2%)
**Start Year**			
2003-2009	9 (40.9%)	1 (4.2%)	3 (33.3%)
2010-2013	4 (18.2%)	9 (37.5%)	0 (0%)
2014-2019	2 (9.1%)	7 (29.2%)	1 (11.1%)
Unspecified	7 (31.8%)	7 (29.2%)	5 (55.6%)
**End Year**			
2009-2012	11 (50%)	4 (16.7%)	3 (33.3%)
2013-2016	2 (9.1%)	11 (45.8%)	0 (0%)
2017-2020	2 (9.1%)	2 (8.3%)	1 (11.1%)
Unspecified	7 (31.8%)	7 (29.2%)	5 (55.6%)
**Lab Country**			
United States	15 (68.2%)	10 (41.7%)	1 (11.1%)
China	0 (0%)	5 (20.8%)	2 (22.2%)
South Korea	0 (0%)	3 (12.5%)	0 (0%)
Cyprus	2 (9.1%)	0 (0%)	1 (11.1%)
France	1 (4.5%)	2 (8.3%)	0 (0%)
Germany	0 (0%)	2 (8.3%)	0 (0%)
Hong Kong	0 (0%)	0 (0%)	2 (22.2%)
UK	2 (9.1%)	0 (0%)	0 (0%)
Sweden	0 (0%)	0 (0%)	2 (22.2%)
Switzerland	0 (0%)	0 (0%)	1 (11.1%)
Thailand	1 (4.5%)	0 (0%)	0 (0%)
Australia	1 (4.5%)	0 (0%)	0 (0%)
Japan	0 (0%)	1 (4.2%)	0 (0%)
Malasia, Canada	0 (0%)	1 (4.2%)	0 (0%)

	Retrospective	Prospective	Other
**Population Country**			
UK	5 (22.7%)	1 (4.2%)	1 (11.1%)
International	4 (18.2%)	2 (8.3%)	0 (0%)
China	0 (0%)	5 (20.8%)	1 (11.1%)
United States	2 (9.1%)	2 (8.3%)	1 (11.1%)
France	1 (4.5%)	2 (8.3%)	0 (0%)
South Korea	0 (0%)	2 (8.3%)	0 (0%)
Japan	0 (0%)	1 (4.2%)	1 (11.1%)
HK, Netherlands, UK	0 (0%)	0 (0%)	2 (22.2%)
US, Netherlands, Sweden	0 (0%)	1 (4.2%)	0 (0%)
UK, US	1 (4.5%)	0 (0%)	0 (0%)
UK, Sweden, France	0 (0%)	0 (0%)	1 (11.1%)
Thailand	1 (4.5%)	0 (0%)	0 (0%)
Taiwan	0 (0%)	1 (4.2%)	0 (0%)
Sweden, UK, US	1 (4.5%)	0 (0%)	0 (0%)
Netherlands, Sweden	0 (0%)	1 (4.2%)	0 (0%)
Malasia, Canada	0 (0%)	1 (4.2%)	0 (0%)
Italy	0 (0%)	1 (4.2%)	0 (0%)
Greece/Cyprus	1 (4.5%)	0 (0%)	0 (0%)
Germany/Switzerland	0 (0%)	1 (4.2%)	0 (0%)
Germany	0 (0%)	1 (4.2%)	0 (0%)
Denmark	0 (0%)	1 (4.2%)	0 (0%)
Canada	0 (0%)	1 (4.2%)	0 (0%)
Australia	1 (4.5%)	0 (0%)	0 (0%)
Unspecified	5 (22.7%)	0 (0%)	2 (22.2%)
**Population Country**			
United States Only	2 (9.1%)	2 (8.3%)	1 (11.1%)
Europe Only	2 (9.1%)	6 (25%)	0 (0%)
International	11 (50%)	5 (20.8%)	5 (55.6%)
Other	7 (31.8%)	11 (45.8%)	3 (33.3%)

	Retrospective	Prospective	Other
**Gestational Age**			
Median (25%ile, 75%ile)	15 (13, 15.4)	15.3 (12.7, 16.6)	13.1 (12.8, 13.8)
96% at 15-20wks	0 (0%)	1 (4.2%)	0 (0%)
Approx 12 - 14.9	0 (0%)	1 (4.2%)	0 (0%)
Approx 19	0 (0%)	1 (4.2%)	0 (0%)
Range 11 - 13.9	1 (4.5%)	0 (0%)	0 (0%)
Unspecified	1 (4.5%)	1 (4.2%)	3 (33.3%)
**Maternal Age**			
Median (25%ile, 75%ile)	35.1 (33.4, 36)	35 (32.3, 36)	35.4 (34.1, 36.6)
88% <35	0 (0%)	1 (4.2%)	0 (0%)
Unspecified	2 (9.1%)	1 (4.2%)	3 (33.3%)
**Major Study Groups**			
High risk: FTS, STS, u/s, AMA	9 (40.9%)	13 (54.2%)	1 (11.1%)
High +Low risk groups combined	6 (27.3%)	1 (4.2%)	0 (0%)
Known aneuploid & euploid mix	1 (4.5%)	1 (4.2%)	5 (55.6%)
All risk	1 (4.5%)	4 (16.7%)	0 (0%)
Unspecified	1 (4.5%)	0 (0%)	1 (11.1%)
<35	0 (0%)	1 (4.2%)	0 (0%)
>=35	0 (0%)	1 (4.2%)	0 (0%)
Combined test risk >=1000	0 (0%)	1 (4.2%)	0 (0%)
High risk + mixed risk	0 (0%)	0 (0%)	1 (11.1%)
High risk: FTS	0 (0%)	1 (4.2%)	0 (0%)
High risk: FTS, u/s, AMA	0 (0%)	1 (4.2%)	0 (0%)
Mainly high risk	1 (4.5%)	0 (0%)	0 (0%)
Prior NIPT with confirmation	1 (4.5%)	0 (0%)	0 (0%)
**Method**			
MPSS	12 (54.5%)	12 (50%)	5 (55.6%)
DANSR	6 (27.3%)	5 (20.8%)	2 (22.2%)
SNP	3 (13.6%)	3 (12.5%)	0 (0%)
circle-amp	0 (0%)	2 (8.3%)	2 (22.2%)

	**Retrospective**	**Prospective**	**Other**
Semi-Conductor	0 (0%)	2 (8.3%)	0 (0%)
MeDIP	1 (4.5%)	0 (0%)	0 (0%)

Supplemental Table S4. See attached Excel spreadsheet.

Supplemental Table S5. Mean Diagnostic Odds Ratios (DORs) stratified by study design

Syndrome	Type	N Studies	Mean DOR (95% CI)
T21	Retrospective	16	31070 (14245, 67768)
	Prospective	24	36699 (19474, 69160)
T18	Retrospective	13	10297 (4385, 24180)
	Prospective	23	10191 (5516, 18828)
T13	Retrospective	12	4986 (2042, 12176)
	Prospective	21	7783 (3547, 17077)
MX	Retrospective	9	2101 (570, 7749)
	Prospective	8	1627 (478, 5532)

Supplemental Table S6. Summary of clinical study characteristics by NIPT methodology.

	MPSS	DANSR	SNP
**N**	**55**	**12**	**12**
**Publication Year**			
2012-2014	4 (7.3%)	4 (33.3%)	2 (16.7%)
2015-2018	8 (14.5%)	3 (25%)	6 (50%)
2019-2022	43 (78.2%)	5 (41.7%)	4 (33.3%)
**Start Year**			
2010-2013	14 (25.5%)	6 (50%)	9 (75%)
2014-2019	35 (63.6%)	5 (41.7%)	2 (16.7%)
Unspecified	6 (10.9%)	1 (8.3%)	1 (8.3%)
**End Year**			
2012-2016	13 (23.6%)	7 (58.3%)	8 (66.7%)
2017-2020	41 (74.5%)	5 (41.7%)	4 (33.3%)
Unspecified	1 (1.8%)	0 (0%)	0 (0%)
**Lab Country**			

	MPSS	DANSR	SNP
China	32 (58.2%)	0 (0%)	0 (0%)
United States	7 (12.7%)	9 (75%)	12 (100%)
Italy	4 (7.3%)	1 (8.3%)	0 (0%)
Switzerland	2 (3.6%)	0 (0%)	0 (0%)
South Korea	1 (1.8%)	0 (0%)	0 (0%)
Netherlands	2 (3.6%)	0 (0%)	0 (0%)
Germany	2 (3.6%)	0 (0%)	0 (0%)
UK	2 (3.6%)	0 (0%)	0 (0%)
Turkey	0 (0%)	1 (8.3%)	0 (0%)
Saudi Arabia	1 (1.8%)	0 (0%)	0 (0%)
Japan	0 (0%)	0 (0%)	0 (0%)
Hong Kong	1 (1.8%)	0 (0%)	0 (0%)
Cyprus	0 (0%)	1 (8.3%)	0 (0%)
France	0 (0%)	0 (0%)	0 (0%)
Unspecified	3 (5.5%)	1 (8.3%)	2 (16.7%)
**Population Country**			
China	31 (56.4%)	0 (0%)	0 (0%)
United States	3 (5.5%)	2 (16.7%)	1 (8.3%)
Italy	4 (7.3%)	0 (0%)	0 (0%)
UK	2 (3.6%)	2 (16.7%)	0 (0%)
Spain	0 (0%)	3 (25%)	1 (8.3%)
Germany	2 (3.6%)	1 (8.3%)	0 (0%)
Thailand	1 (1.8%)	0 (0%)	1 (8.3%)
Switzerland	1 (1.8%)	0 (0%)	1 (8.3%)
South Korea	1 (1.8%)	0 (0%)	0 (0%)
Netherlands	2 (3.6%)	0 (0%)	0 (0%)
International	2 (3.6%)	0 (0%)	0 (0%)
Turkey	0 (0%)	1 (8.3%)	0 (0%)
Spain/Portugal	0 (0%)	0 (0%)	1 (8.3%)

	MPSS	DANSR	SNP
Saudi Arabia	1 (1.8%)	0 (0%)	0 (0%)
Russia	0 (0%)	0 (0%)	1 (8.3%)
Romania	0 (0%)	0 (0%)	1 (8.3%)
Lithuania	0 (0%)	0 (0%)	1 (8.3%)
Japan	1 (1.8%)	0 (0%)	0 (0%)
Iran	1 (1.8%)	0 (0%)	0 (0%)
India	0 (0%)	0 (0%)	1 (8.3%)
France	0 (0%)	0 (0%)	0 (0%)
Denmark	0 (0%)	0 (0%)	0 (0%)
Belgium/Netherlands	0 (0%)	1 (8.3%)	0 (0%)
Austria/Germany	0 (0%)	0 (0%)	1 (8.3%)
Germany & others	0 (0%)	1 (8.3%)	0 (0%)
**Gestational Age**			
Median (25%ile, 75%ile)	15.6 (13.2, 17.4)	12.9 (12.6, 13)	13.5 (12.3, 14.2)
Unspecified	10 (18.2%)	0 (0%)	1 (8.3%)
**Maternal Age**			
Median (25%ile, 75%ile)	33 (30.7, 35.1)	34.5 (32.3, 36.2)	35 (34.7, 36.7)
Unspecified	15 (27.3%)	0 (0%)	1 (8.3%)
**Major Study Groups**			
All risk	32 (58.2%)	6 (50%)	5 (41.7%)
Mostly high risk	8 (14.5%)	0 (0%)	4 (33.3%)
High risk, exclud. Abn u/s	1 (0%)	2 (0%)	1 (0%)
All except abn u/s	0 (0%)	1 (8.3%)	1 (8.3%)
Primary & Secondary	1 (1.8%)	0 (0%)	0 (0%)
NT< 3.5mm	0 (0%)	1 (8.3%)	0 (0%)
NL FTS. Soft u/s markers present	1 (1.8%)	0 (0%)	0 (0%)
Mostly AMA	1 (1.8%)	0 (0%)	1 (8.3%)
Mixed	0 (0%)	0 (0%)	0 (0%)
Mat age >=35	1 (1.8%)	0 (0%)	0 (0%)

	**MPSS**	**DANSR**	**SNP**
High risk: FTS, STS, u/s, AMA	1 (1.8%)	0 (0%)	0 (0%)
High risk: FTS, STS	1 (1.8%)	0 (0%)	0 (0%)
High risk, mostly exclud. Abn u/s	1 (1.8%)	0 (0%)	0 (0%)
High or intermdiate risk: FTS, STS, excl u/s abn	1 (1.8%)	0 (0%)	0 (0%)
FTS 1in 50 to 1 in 270	0 (0%)	1 (8.3%)	0 (0%)
First trimester screen positive	1 (1.8%)	0 (0%)	0 (0%)
All risk, ecl abn u/s	1 (1.8%)	0 (0%)	0 (0%)

Supplemental Table S7. See attached Excel spreadsheet.

Supplemental Table S8. Number and percentage of high-risk results where there was follow-up

Syndrome	Method	# HR Calls	# Confirmed	Percent Confirmed
T21	All	17424	7771	44.60
	MPSS	8375	6061	72.37
	DANSR	279	193	69.18
	SNP	8584	1337	15.58
T18	All	5289	2448	46.28
	MPSS	2778	1916	68.97
	DANSR	62	44	70.97
	SNP	2369	413	17.43
T13	All	2694	1175	43.62
	MPSS	1308	930	71.10
	DANSR	38	24	63.16
	SNP	1311	184	14.04
MX	All	4039	1652	40.90
	MPSS	1840	1416	76.96
	DANSR	79	70	88.61
	SNP	2120	166	7.83

Supplemental Figure S1 Forest plot data

T21 Sensitivity

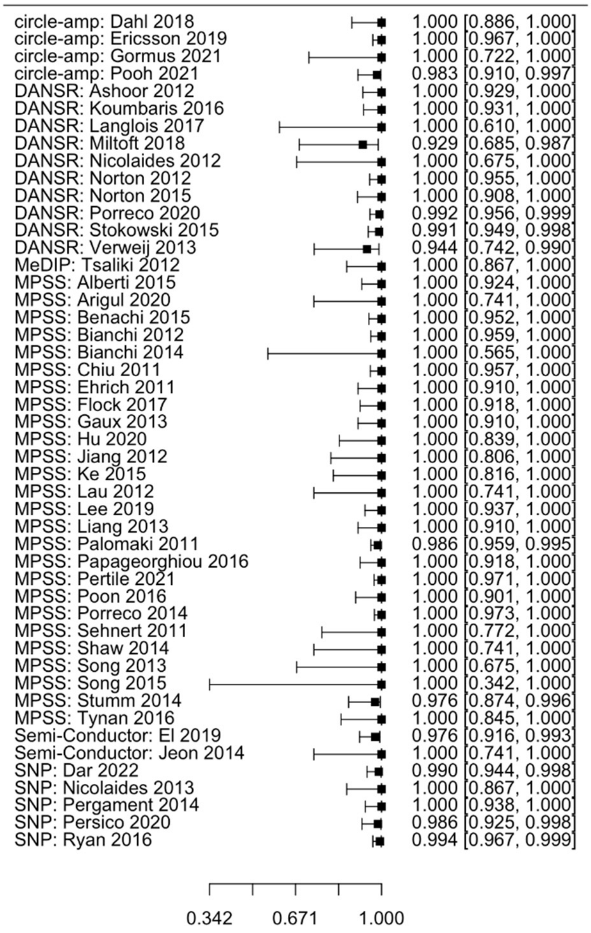

T21 Specificity

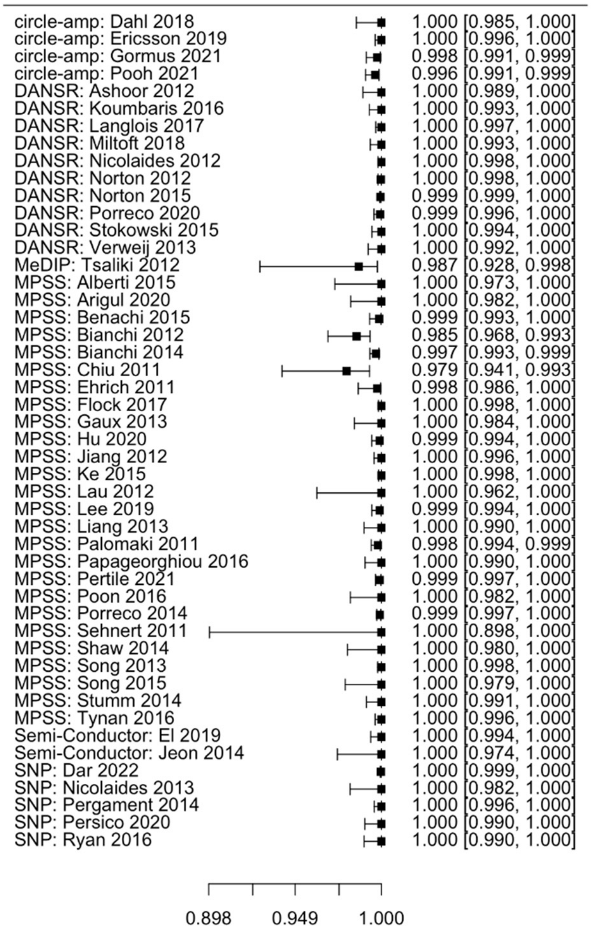

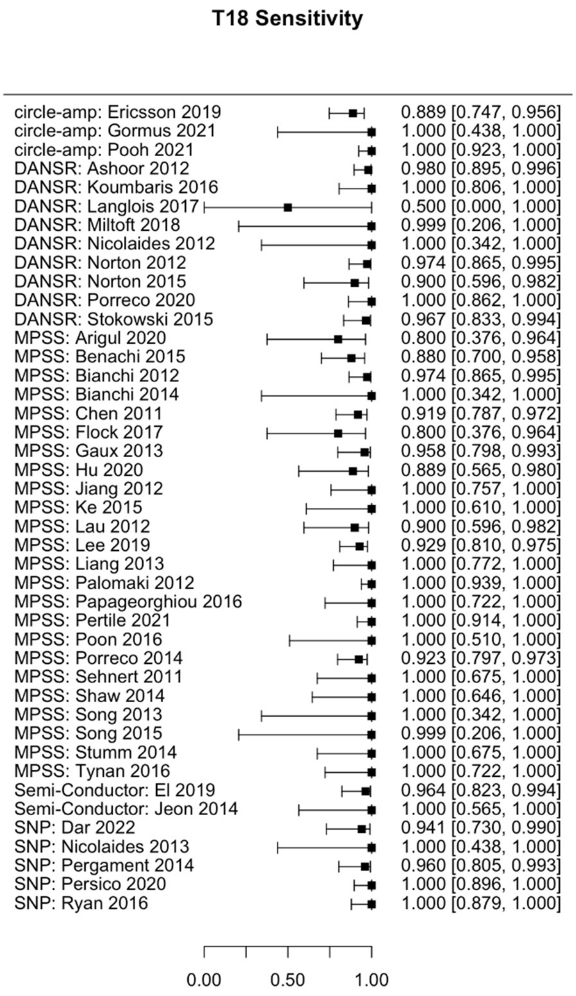
T18 Sensitivity
circle-amp: Ericsson 2019 0.889 [0.747, 0.956]
circle-amp: Gormus 2021 1.000 [0.438, 1.000]
circle-amp: Pooh 2021 1.000 [0.923, 1.000]
DANSR: Ashoor 2012 0.980 [0.895, 0.996]
DANSR: Koumbaris 2016 1.000 [0.806, 1.000]
DANSR: Langlois 2017 0.500 [0.000, 1.000]
DANSR: Miltoft 2018 0.999 [0.206, 1.000]
DANSR: Nicolaides 2012 1.000 [0.342, 1.000]
DANSR: Norton 2012 0.974 [0.865, 0.995]
DANSR: Norton 2015 0.900 [0.596, 0.982]
DANSR: Porreco 2020 1.000 [0.862, 1.000]
DANSR: Stokowski 2015 0.967 [0.833, 0.994]
MPSS: Arigul 2020 0.800 [0.376, 0.964]
MPSS: Benachi 2015 0.880 [0.700, 0.958]
MPSS: Bianchi 2012 0.974 [0.865, 0.995]
MPSS: Bianchi 2014 1.000 [0.342, 1.000]
MPSS: Chen 2011 0.919 [0.787, 0.972]
MPSS: Flock 2017 0.800 [0.376, 0.964]
MPSS: Gaux 2013 0.958 [0.798, 0.993]
MPSS: Hu 2020 0.889 [0.565, 0.980]
MPSS: Jiang 2012 1.000 [0.757, 1.000]
MPSS: Ke 2015 1.000 [0.610, 1.000]
MPSS: Lau 2012 0.900 [0.596, 0.982]
MPSS: Lee 2019 0.929 [0.810, 0.975]
MPSS: Liang 2013 1.000 [0.772, 1.000]
MPSS: Palomaki 2012 1.000 [0.939, 1.000]
MPSS: Papageorghiou 2016 1.000 [0.722, 1.000]
MPSS: Pertile 2021 1.000 [0.914, 1.000]
MPSS: Poon 2016 1.000 [0.510, 1.000]
MPSS: Porreco 2014 0.923 [0.797, 0.973]
MPSS: Sehnert 2011 1.000 [0.675, 1.000]
MPSS: Shaw 2014 1.000 [0.646, 1.000]
MPSS: Song 2013 1.000 [0.342, 1.000]
MPSS: Song 2015 0.999 [0.206, 1.000]
MPSS: Stumm 2014 1.000 [0.675, 1.000]
MPSS: Tynan 2016 1.000 [0.722, 1.000]
Semi-Conductor: El 2019 0.964 [0.823, 0.994]
Semi-Conductor: Jeon 2014 1.000 [0.565, 1.000]
SNP: Dar 2022 0.941 [0.730, 0.990]
SNP: Nicolaides 2013 1.000 [0.438, 1.000]
SNP: Pergament 2014 0.960 [0.805, 0.993]
SNP: Persico 2020 1.000 [0.896, 1.000]
SNP: Ryan 2016 1.000 [0.879, 1.000]
0.00
0.50
1.00

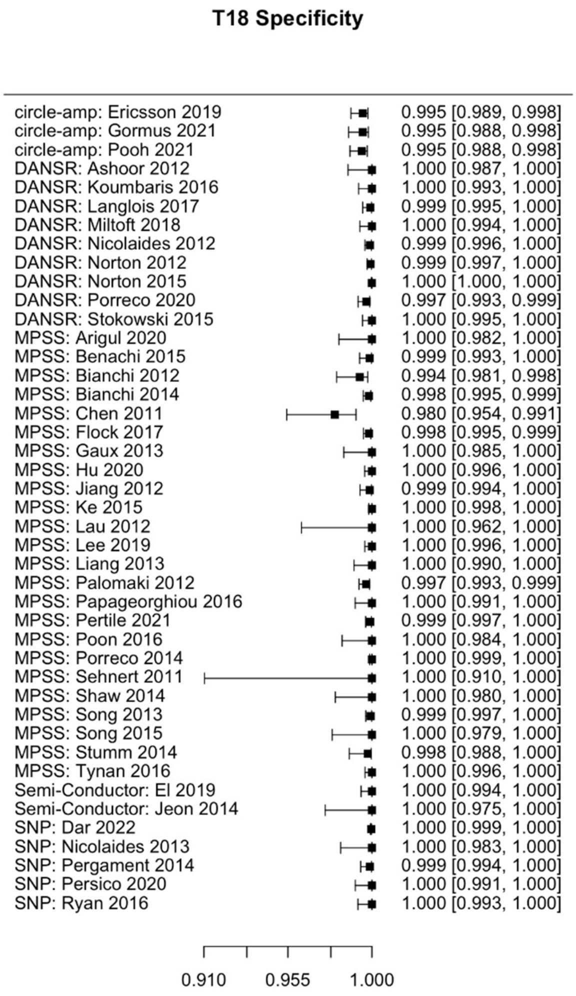
T18 Specificity
circle-amp: Ericsson 2019 0.995 [0.989, 0.998]
circle-amp: Gormus 2021 0.995 [0.988, 0.998]
circle-amp: Pooh 2021 0.995 [0.988, 0.998]
DANSR: Ashoor 2012 1.000 [0.987, 1.000]
DANSR: Koumbaris 2016 1.000 [0.993, 1.000]
DANSR: Langlois 2017 0.999 [0.995, 1.000]
DANSR: Miltoft 2018 1.000 [0.994, 1.000]
DANSR: Nicolaides 2012 0.999 [0.996, 1.000]
DANSR: Norton 2012 0.999 [0.997, 1.000]
DANSR: Norton 2015 1.000 [1.000, 1.000]
DANSR: Porreco 2020 0.997 [0.993, 0.999]
DANSR: Stokowski 2015 1.000 [0.995, 1.000]
MPSS: Arigul 2020 1.000 [0.982, 1.000]
MPSS: Benachi 2015 0.999 [0.993, 1.000]
MPSS: Bianchi 2012 0.994 [0.981, 0.998]
MPSS: Bianchi 2014 0.998 [0.995, 0.999]
MPSS: Chen 2011 0.980 [0.954, 0.991]
MPSS: Flock 2017 0.998 [0.995, 0.999]
MPSS: Gaux 2013 1.000 [0.985, 1.000]
MPSS: Hu 2020 1.000 [0.996, 1.000]
MPSS: Jiang 2012 0.999 [0.994, 1.000]
MPSS: Ke 2015 1.000 [0.998, 1.000]
MPSS: Lau 2012 1.000 [0.962, 1.000]
MPSS: Lee 2019 1.000 [0.996, 1.000]
MPSS: Liang 2013 1.000 [0.990, 1.000]
MPSS: Palomaki 2012 0.997 [0.993, 0.999]
MPSS: Papageorghiou 2016 1.000 [0.991, 1.000]
MPSS: Pertile 2021 0.999 [0.997, 1.000]
MPSS: Poon 2016 1.000 [0.984, 1.000]
MPSS: Porreco 2014 1.000 [0.999, 1.000]
MPSS: Sehnert 2011 1.000 [0.910, 1.000]
MPSS: Shaw 2014 1.000 [0.980, 1.000]
MPSS: Song 2013 0.999 [0.997, 1.000]
MPSS: Song 2015 1.000 [0.979, 1.000]
MPSS: Stumm 2014 0.998 [0.988, 1.000]
MPSS: Tynan 2016 1.000 [0.996, 1.000]
Semi-Conductor: El 2019 1.000 [0.994, 1.000]
Semi-Conductor: Jeon 2014 1.000 [0.975, 1.000]
SNP: Dar 2022 1.000 [0.999, 1.000]
SNP: Nicolaides 2013 1.000 [0.983, 1.000]
SNP: Pergament 2014 0.999 [0.994, 1.000]
SNP: Persico 2020 1.000 [0.991, 1.000]
SNP: Ryan 2016 1.000 [0.993, 1.000]
0.910
0.955
1.000

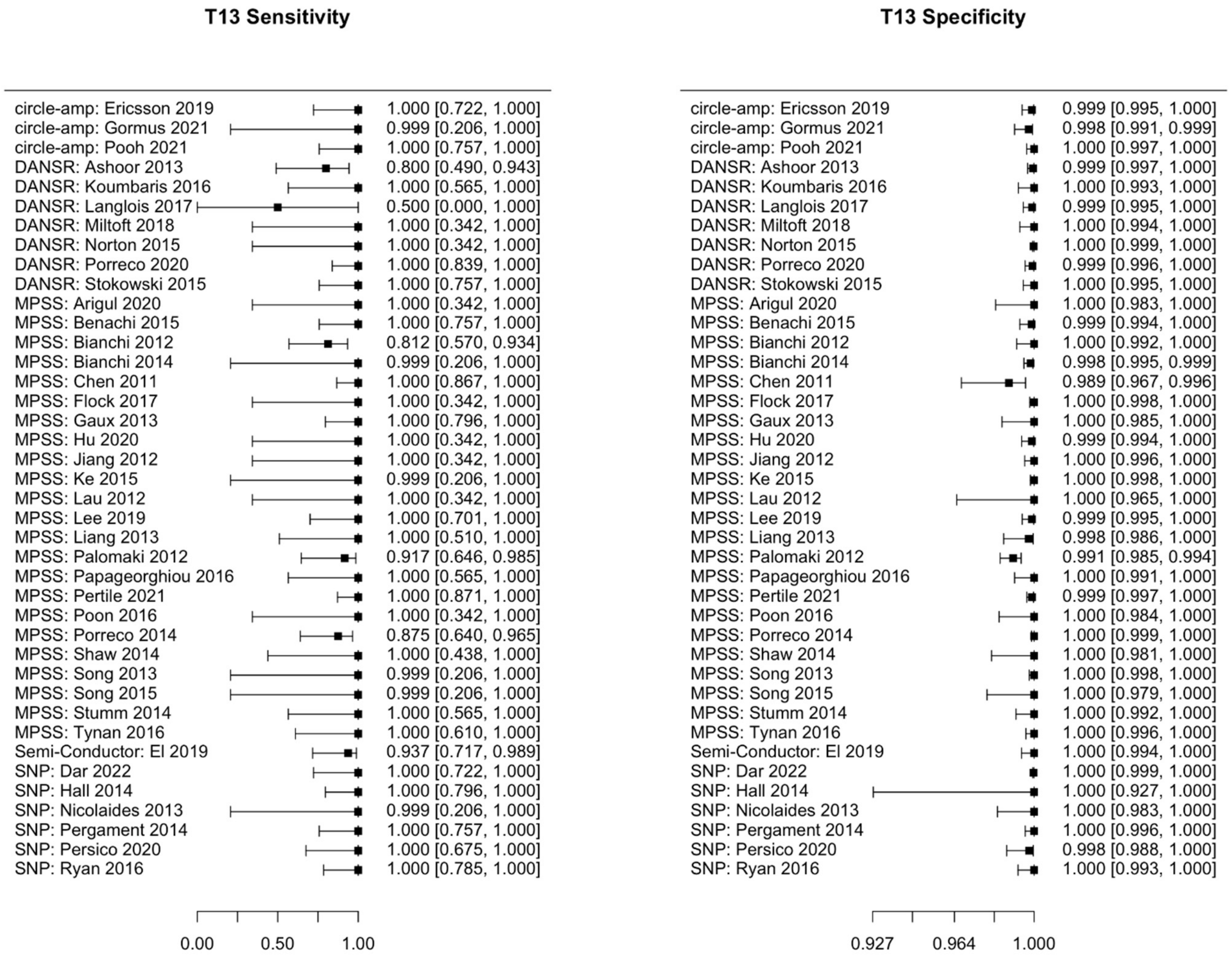
T13 Sensitivity
circle-amp: Ericsson 2019 1.000 [0.722, 1.000]
circle-amp: Gormus 2021 0.999 [0.206, 1.000]
circle-amp: Pooh 2021 1.000 [0.757, 1.000]
DANSR: Ashoor 2013 0.800 [0.490, 0.943]
DANSR: Koumbaris 2016 1.000 [0.565, 1.000]
DANSR: Langlois 2017 0.500 [0.000, 1.000]
DANSR: Miltoft 2018 1.000 [0.342, 1.000]
DANSR: Norton 2015 1.000 [0.342, 1.000]
DANSR: Porreco 2020 1.000 [0.839, 1.000]
DANSR: Stokowski 2015 1.000 [0.757, 1.000]
MPSS: Arigul 2020 1.000 [0.342, 1.000]
MPSS: Benachi 2015 1.000 [0.757, 1.000]
MPSS: Bianchi 2012 0.812 [0.570, 0.934]
MPSS: Bianchi 2014 0.999 [0.206, 1.000]
MPSS: Chen 2011 1.000 [0.867, 1.000]
MPSS: Flock 2017 1.000 [0.342, 1.000]
MPSS: Gaux 2013 1.000 [0.796, 1.000]
MPSS: Hu 2020 1.000 [0.342, 1.000]
MPSS: Jiang 2012 1.000 [0.342, 1.000]
MPSS: Ke 2015 0.999 [0.206, 1.000]
MPSS: Lau 2012 1.000 [0.342, 1.000]
MPSS: Lee 2019 1.000 [0.701, 1.000]
MPSS: Liang 2013 1.000 [0.510, 1.000]
MPSS: Palomaki 2012 0.917 [0.646, 0.985]
MPSS: Papageorghiou 2016 1.000 [0.565, 1.000]
MPSS: Pertile 2021 1.000 [0.871, 1.000]
MPSS: Poon 2016 1.000 [0.342, 1.000]
MPSS: Porreco 2014 0.875 [0.640, 0.965]
MPSS: Shaw 2014 1.000 [0.438, 1.000]
MPSS: Song 2013 0.999 [0.206, 1.000]
MPSS: Song 2015 0.999 [0.206, 1.000]
MPSS: Stumm 2014 1.000 [0.565, 1.000]
MPSS: Tynan 2016 1.000 [0.610, 1.000]
Semi-Conductor: El 2019 0.937 [0.717, 0.989]
SNP: Dar 2022 1.000 [0.722, 1.000]
SNP: Hall 2014 1.000 [0.796, 1.000]
SNP: Nicolaides 2013 0.999 [0.206, 1.000]
SNP: Pergament 2014 1.000 [0.757, 1.000]
SNP: Persico 2020 1.000 [0.675, 1.000]
SNP: Ryan 2016 1.000 [0.785, 1.000]
0.00 0.50 1.00
T13 Specificity
circle-amp: Ericsson 2019 0.999 [0.995, 1.000]
circle-amp: Gormus 2021 0.998 [0.991, 0.999]
circle-amp: Pooh 2021 1.000 [0.997, 1.000]
DANSR: Ashoor 2013 0.999 [0.997, 1.000]
DANSR: Koumbaris 2016 1.000 [0.993, 1.000]
DANSR: Langlois 2017 0.999 [0.995, 1.000]
DANSR: Miltoft 2018 1.000 [0.994, 1.000]
DANSR: Norton 2015 1.000 [0.999, 1.000]
DANSR: Porreco 2020 0.999 [0.996, 1.000]
DANSR: Stokowski 2015 1.000 [0.995, 1.000]
MPSS: Arigul 2020 1.000 [0.983, 1.000]
MPSS: Benachi 2015 0.999 [0.994, 1.000]
MPSS: Bianchi 2012 1.000 [0.992, 1.000]
MPSS: Bianchi 2014 0.998 [0.995, 0.999]
MPSS: Chen 2011 0.989 [0.967, 0.996]
MPSS: Flock 2017 1.000 [0.998, 1.000]
MPSS: Gaux 2013 1.000 [0.985, 1.000]
MPSS: Hu 2020 0.999 [0.994, 1.000]
MPSS: Jiang 2012 1.000 [0.996, 1.000]
MPSS: Ke 2015 1.000 [0.998, 1.000]
MPSS: Lau 2012 1.000 [0.965, 1.000]
MPSS: Lee 2019 0.999 [0.995, 1.000]
MPSS: Liang 2013 0.998 [0.986, 1.000]
MPSS: Palomaki 2012 0.991 [0.985, 0.994]
MPSS: Papageorghiou 2016 1.000 [0.991, 1.000]
MPSS: Pertile 2021 0.999 [0.997, 1.000]
MPSS: Poon 2016 1.000 [0.984, 1.000]
MPSS: Porreco 2014 1.000 [0.999, 1.000]
MPSS: Shaw 2014 1.000 [0.981, 1.000]
MPSS: Song 2013 1.000 [0.998, 1.000]
MPSS: Song 2015 1.000 [0.979, 1.000]
MPSS: Stumm 2014 1.000 [0.992, 1.000]
MPSS: Tynan 2016 1.000 [0.996, 1.000]
Semi-Conductor: El 2019 1.000 [0.994, 1.000]
SNP: Dar 2022 1.000 [0.999, 1.000]
SNP: Hall 2014 1.000 [0.927, 1.000]
SNP: Nicolaides 2013 1.000 [0.983, 1.000]
SNP: Pergament 2014 1.000 [0.996, 1.000]
SNP: Persico 2020 0.998 [0.988, 1.000]
SNP: Ryan 2016 1.000 [0.993, 1.000]
0.927 0.964 1.000

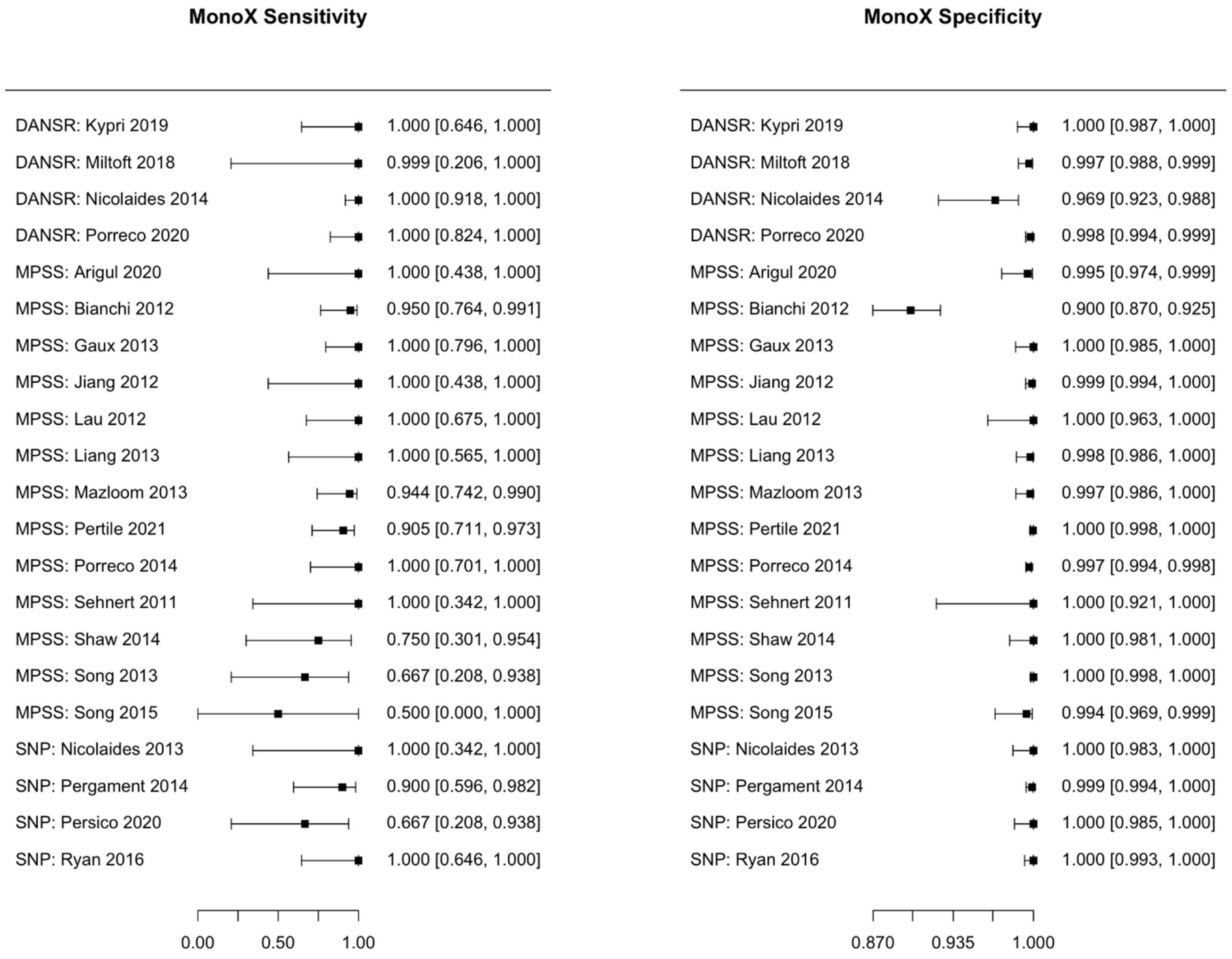
MonoX Sensitivity
DANSR: Kypri 2019 1.000 [0.646, 1.000]
DANSR: Miltoft 2018 0.999 [0.206, 1.000]
DANSR: Nicolaides 2014 1.000 [0.918, 1.000]
DANSR: Porreco 2020 1.000 [0.824, 1.000]
MPSS: Arigul 2020 1.000 [0.438, 1.000]
MPSS: Bianchi 2012 0.950 [0.764, 0.991]
MPSS: Gaux 2013 1.000 [0.796, 1.000]
MPSS: Jiang 2012 1.000 [0.438, 1.000]
MPSS: Lau 2012 1.000 [0.675, 1.000]
MPSS: Liang 2013 1.000 [0.565, 1.000]
MPSS: Mazloom 2013 0.944 [0.742, 0.990]
MPSS: Pertile 2021 0.905 [0.711, 0.973]
MPSS: Porreco 2014 1.000 [0.701, 1.000]
MPSS: Sehnert 2011 1.000 [0.342, 1.000]
MPSS: Shaw 2014 0.750 [0.301, 0.954]
MPSS: Song 2013 0.667 [0.208, 0.938]
MPSS: Song 2015 0.500 [0.000, 1.000]
SNP: Nicolaides 2013 1.000 [0.342, 1.000]
SNP: Pergament 2014 0.900 [0.596, 0.982]
SNP: Persico 2020 0.667 [0.208, 0.938]
SNP: Ryan 2016 1.000 [0.646, 1.000]
0.00
0.50
1.00
MonoX Specificity
DANSR: Kypri 2019 1.000 [0.987, 1.000]
DANSR: Miltoft 2018 0.997 [0.988, 0.999]
DANSR: Nicolaides 2014 0.969 [0.923, 0.988]
DANSR: Porreco 2020 0.998 [0.994, 0.999]
MPSS: Arigul 2020 0.995 [0.974, 0.999]
MPSS: Bianchi 2012 0.900 [0.870, 0.925]
MPSS: Gaux 2013 1.000 [0.985, 1.000]
MPSS: Jiang 2012 0.999 [0.994, 1.000]
MPSS: Lau 2012 1.000 [0.963, 1.000]
MPSS: Liang 2013 0.998 [0.986, 1.000]
MPSS: Mazloom 2013 0.997 [0.986, 1.000]
MPSS: Pertile 2021 1.000 [0.998, 1.000]
MPSS: Porreco 2014 0.997 [0.994, 0.998]
MPSS: Sehnert 2011 1.000 [0.921, 1.000]
MPSS: Shaw 2014 1.000 [0.981, 1.000]
MPSS: Song 2013 1.000 [0.998, 1.000]
MPSS: Song 2015 0.994 [0.969, 0.999]
SNP: Nicolaides 2013 1.000 [0.983, 1.000]
SNP: Pergament 2014 0.999 [0.994, 1.000]
SNP: Persico 2020 1.000 [0.985, 1.000]
SNP: Ryan 2016 1.000 [0.993, 1.000]
0.870
0.935
1.000
